# Wealth-related inequality in effective coverage of postnatal newborn care in Tanzania; A decomposition analysis of the Tanzania Demographic and Health Survey, 2022

**DOI:** 10.1371/journal.pone.0358220

**Published:** 2026-09-17

**Authors:** Amanuel Yosef Gebrekidan, Yordanos Sisay Asgedom, Mesfin Abebe, Tsion Mulat Tebeje, Kassahun Animut Metkie, Simegn Molla Degisew, Kirubel Eshetu Haile, Seblewongel Gebretsadik Sertsewold, Gizachew Ambaw Kassie

**Affiliations:** 1 School of Public Health, College of Health Sciences and Medicine, Wolaita Sodo University, Wolaita Sodo, Ethiopia; 2 School of Nursing and Midwifery, Edith Cowan University, Perth, Western Australia, Australia; 3 Department of Midwifery, College of Health Sciences and Medicine, Dilla University, Dilla, Ethiopia; 4 School of Public Health, College of Health Sciences and Medicine, Dilla University, Dilla, Ethiopia; 5 Department of Statistics, College of Natural and Computational Sciences, Dilla University, Dilla, Ethiopia; 6 School of Nursing, College of Health Sciences and Medicine, Wolaita Sodo University, Wolaita Sodo, Ethiopia; 7 School of Public Health, Asrat Woldeyes Health Science Campus, Debrebirhan University, Debrebirhan, Ethiopia; 8 School of Business, Law, Humanities and Pathways (BLHP) University of Southern Queensland, Toowoomba, Queensland, Australia; Hijli College, INDIA

## Abstract

**Background:**

Effective postnatal newborn care provision is proven to be one effective strategy to reduce neonatal and under-five mortality. Despite the presence of small-scale studies that reported crude coverage of postnatal newborn care, nationwide evidence about effective postnatal newborn care coverage and evidence regarding the status of wealth-related inequality are limited in Tanzania. Therefore, this study aimed to assess the status of wealth-related inequality and determinants in effective coverage of postnatal newborn care in Tanzania.

**Methods:**

A secondary data analysis was conducted based on cross-sectional data from the 2022 Tanzania Demographic and Health Survey. 4335 weighted samples of reproductive age-women (15–49) who had given birth in the two years preceding the survey were included. The Erreygers normalized concentration index and graph approach were employed to evaluate wealth-related inequality in effective coverage of postnatal newborn care. Additionally, a decomposition analysis was used to decompose the overall wealth-related inequality by determinant factors.

**Results:**

The proportion of newborns who received effective postnatal newborn care interventions in Tanzania was 22.70% [95% CI: 21.48, 23.97]. A significant pro-rich wealth-related inequality in effective coverage of postnatal newborn care, with an Erreygers concentration index value of 0.26 (95% CI: 0.24, 0.29) (P < 0.001), was found. The decomposition result showed the most significant factors leading to the overall inequality were place of delivery (68.43%), residence (22.72%), mothers’ educational level (11.62%), region (6.84%), antenatal care follow-up (4.49), and mode of delivery (3.37%).

**Conclusion:**

The study found a low level of effective postnatal newborn care coverage, with a pro-rich distribution. Focus should be given on increasing institutional deliveries, expanding education, promoting ANC follow-up, improving wealth status, and newborns from vaginal deliveries. Furthermore, authorities and stakeholders should work on expanding health services in rural and affected regions to increase the population’s access to newborn health services.

## Introduction

The third goal of the Sustainable Development Goals (SDGs) 2030 Agenda for Sustainable Development is to “ensure healthy lives and promote well-being for all at all ages.” One of the goals is to end preventable newborns and child mortality as well as attain universal health coverage [1]. Although significant progress has been made in lowering neonatal mortality since 1990, additional measures to accelerate development are required to meet the SDG objective of reducing NMR to 12 deaths per 1000 livebirths or fewer by 2030 [2]. Rapid progress is primarily needed in regions and nations with high NMR, particularly SSA and South Asia [2]. According to a projected analysis, if neonatal death reduction rates do not accelerate, 1.8 million neonates will die by 2030. Achieving the SDG objective for each country by 2030 will result in a reduction of newborn deaths by 600,000 [2].

The neonatal period (first 28 days of life) has the highest mortality rate per day compared to other periods of childhood [3]. As a result, for decades, neonatal health and survival have been a key public health priority [4]. Premature birth, birth asphyxia/trauma, neonatal infections, and congenital defects are the top causes of neonatal death [5]. 2.3 million newborn deaths occurred in 2022 globally, and despite the decline in the number of neonatal deaths by 44% since 2000, nearly half (47%) of all deaths in children under the age of five happened during the newborn period in 2022 [5]. The burden is much higher in Sub-Saharan Africa (SSA) compared to the rest of the world, where 57%, or 2.8 million under five deaths, occurred in this region, despite the region contributing only 30% of the total global live births in 2022 [5]. Consistent with total under-five mortality, Sub-Saharan Africa remains the leading region in neonatal mortality rate (NMR), with 27 deaths per 1000 live births [2,5]. The risk of death in the first month in a child born in SSA is tenfold higher than a child born in a high-income nation [6]. Under-five, infant, and neonatal mortality are still significant public health problems in Tanzania, with the United Nations Children’s Fund data showing a 20.6, 30, and 40.5 neonatal, infant, and under-5 deaths per 1000 live births in 2023, respectively [7].

Nearly 80% of newborn mortality could be averted by targeting the time of delivery with proven high-impact interventions and quality care for small and sick newborns [8]. One strategy to reduce neonatal as well as under-five mortality is provision of postnatal newborn care [9,10]. Post-natal care services are an essential component of maternal, newborn, and child care, and they are critical to achieving the SDGs for reproductive, maternal, and child health, which include aims to end preventable newborn deaths [10]. The World Health Organization (WHO) recommends that all mothers and babies should receive postnatal care within the first 24 hours of birth, regardless of where the birth occurs. The postnatal newborn care includes: thermal protection (e.g., promoting skin-to-skin contact between mother and infant); hygienic umbilical cord and skin care; early and exclusive breastfeeding; screening for signs of serious health problems or need for additional care (e.g., low birth weight, sick, or human immunodeficiency virus infected mother); and preventive treatment (e.g., Bacillus Calmette-Guérin vaccine (BCG) and hepatitis B immunizations, vitamin K, and ocular prophylaxis) [9,10]. Families should be reminded to seek immediate medical care if necessary (risk indicators include feeding issues, or if the newborn has decreased activity, difficulty breathing, a fever, fits or convulsions, or feels cold); register the birth; and bring the baby for timely vaccination [9,10]. One strategic aim of Tanzania’s fifth Health Sector Strategic Plan (2021–2026) is to reduce maternal and neonatal morbidity and mortality by ensuring equal access to health and nutrition services. One stated goal of the strategic plan is to increase the quality of care in reproductive, maternity, newborn, child, and adolescent health [11]. The high rates of preventable death, as well as the poor health and well-being of children, are signs of inadequate access and coverage of life-saving measures and, more broadly, of inadequate social and economic development. In addition to the inadequate social and economic developments, the lack of quality health services, such as newborn care, are all adverse factors [5,12]. Globally, only 68% of newborns had a postnatal health care within the recommended time frame [8]. However, in SSA countries, postnatal neonatal care coverage is low and varies by country, with data indicating coverage of 13.6%, 25.3%, 34.5%, 49.4%, and 83.1% in the Comoros, Gabon, Ethiopia, Burundi, and Cote D’ivoire, respectively [8]. A recent study in Pemba, Tanzania, found that none of the neonates delivered in a health institution received all eight of the recommended essential newborn interventions [13]. The problem is exacerbated by the presence of inequalities in newborn contact coverage and quality of care offered, with evidence showing inequalities based on individual, family, contextual, and structural factors [14–16]. Various studies have indicated wealth-related inequality in effective postnatal newborn care, and this discrepancy is also associated with antenatal care (ANC) [16], age [14], educational status [14,16], maternal age [14], living with a partner [14], and residence [14].

Most studies use crude coverage indicators, which are frequently used in maternal and child health studies, representing the tip of the iceberg because they only consider contact between women or children and health care providers. They are unable to show the level of adherence to care standards, the manner in which services are delivered, or the quality of care offered. On the other hand, effective coverage combines the contact between service users and health services with the quality of care received, providing a proxy estimate of the potential desired health care outcomes through using the services [15,17]. This study assessed whether or not the contact between newborns and healthcare providers resulted in the provision of standard newborn care services, focusing on the process aspect of healthcare quality. Furthermore, nationwide evidence about effective coverage of postnatal newborn care and evidence regarding the presence or absence of wealth-related inequality in relation to effective coverage of newborn care are limited in Tanzania, resulting in the lack of baseline information that can help in the assessment of the implementation and progress of the Health Sector Strategic Plan V. This study will address the following research questions: 1. What is the percentage of newborns in Tanzania who receive effective postnatal care? 2. Is there wealth-related inequality in receiving effective postnatal newborn care in Tanzania, and if so, 3. What factors contribute to the wealth-related inequality? Therefore, this study aimed to examine wealth-related inequality in the effective coverage of postnatal newborn care and its determinants in Tanzania, with the objective of providing baseline information on the national-level effective coverage (quality-adjusted coverage), inequality status, and its determinants, thereby facilitating evidence-based decision-making and supporting the monitoring and evaluation of the Health Sector Strategic Plan V implementation.

## Methods

### Study design, area, and period

We conducted a secondary data analysis based on cross-sectional data from the Tanzania Demographic and Health Survey and Malaria Indicator Survey (2022 TDHS-MIS). Tanzania’s population and housing census for 2022 shows a total population of 61.7 million, with 59,851,347 living on mainland Tanzania and 1,889,773 in Zanzibar. Rural and urban areas represent 40.2 million and 21.5 million of the total population, respectively [18]. The entire population consisted of 31.7 million females and 30 million males [18]. The 2022 TDHS-MIS is the seventh in a series of DHS surveys in Tanzania undertaken by the Demographic and Health Surveys Programme [19]. Previous demographic and health surveys were conducted in 1991–1992, 1996, 1999, 2004–2005, 2010, and 2015–2016 [19]. To do this, the survey used a nationally representative sample of 16,354 households [19]. Tanzania was divided into nine administrative zones to analyze spatial variations in population indicators. The Reproductive and Child Health Section of the Ministry of Health uses this classification scheme, despite the fact that these zones are not formal administrative areas. Grouping regions into zones increases denominators and reduces sampling errors for zonal indicators [19].

### Data source, population and sampling procedure

This analysis was based on the most recent 2022 TDHS-MIS. The 2022 TDHS-MIS sample design was divided into two stages. The first stage comprised selecting sampling points (clusters) from enumeration areas (EAs) chosen for the 2012 Tanzania Population and Housing Census (PHC) [19]. EAs were chosen at a rate proportional to their size within each sampling stratum. In total, 629 clusters were identified. Of the 629 EAs, 211 were from urban and 418 from rural areas. The second stage consisted of choosing 26 households from each cluster, providing a total sample size of 16,354 for the 2022 TDHS-MIS [19].

**Fig 1:**
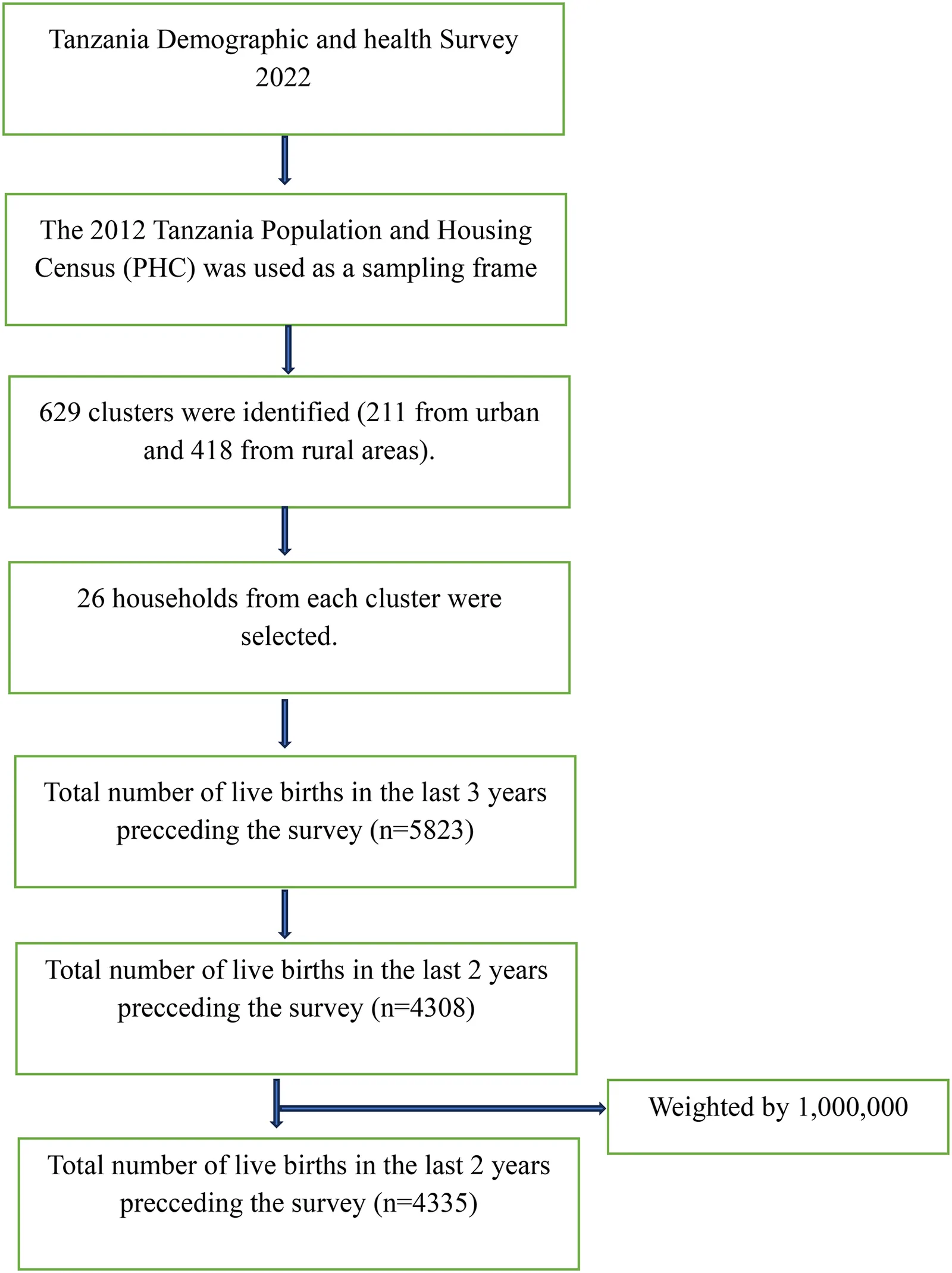
Sampling procedure for wealth-related inequality in effective coverage of postnatal newborn care in Tanzania; A decomposition analysis of the Tanzania Demographic and Health Survey, 2022.

This study used data from the most recent live births of interviewed mothers in the 2 years preceding the survey (NR File). To provide a representative sample, women (v005) were weighted by dividing their individual weight by 1,000,000 to estimate the number of cases. We retrieved data from this large dataset for reproductive age (15–49) who had given birth within the previous two years since the suggested indicators for postnatal care are based on births within the two years preceding the survey [20]. According to the guide to DHS statistics recommendations, most recent live births in the 2 years preceding the survey were included, while live births that occurred prior to the preceding 2 years were excluded [20]. This study included 4335 weighted sample women of reproductive age (15–49) who had given birth in the two years preceding the survey (Fig 1). The Strengthening Reporting of Observational Studies in Epidemiology (STROBE) guideline was followed in the preparation of this manuscript (S1 File) [21].

### Study variables and measurement

#### Outcome variable.

The outcome variable, which is effective coverage, was assessed by choosing seven newborn intervention items: a woman reporting that her baby received the following services: temperature measurement, danger sign counseling, cord examination, breastfeeding counseling, breastfeeding observation, told about child vaccination, and newborn weighed [15,20,22–24]. The response to each question/item was classified as ‘1’ if a specific service was provided and ‘0’ otherwise. Each woman’s “yes” responses were added together to construct an indicator of effective postnatal newborn care [15,22]. It was then defined as the percentage of neonates who received a postnatal check within 48 hours of birth and had all seven of their care needs met. Effective postnatal newborn was classified as “Provided” if the woman scored seven of the seven interventions. Crude (contact) coverage was defined as the proportion of children born alive in the previous two years whose mothers reported receiving a postnatal check within 48 hours of delivery [15].

The wealth-related inequality in effective coverage of postnatal newborn care was then assessed using the covariance between the provision of effective newborn care interventions and the distribution of living standards (wealth index). The wealth status was determined using a wealth index. Household wealth indices were calculated using the amount and kind of consumer goods owned, such as televisions, bicycles, or vehicles, as well as living amenities including, drinking water, bathroom facilities, and flooring materials. It was determined using principal component analysis and grouped into five quintiles: poorest (quintile 1), poorer (quintile 2), middle (quintile 3), richer (quintile 4), and richest (quintile 5) [20].Then it was categorized as either pro-poor, pro-rich, or no inequality.

#### Independent variables.

The independent variables in this study included sociodemographic variables: age of the mother, the sex of the household head, marital status, education level, work status, place of residence, and region; obstetric characteristics: parity, ANC follow-up, number of ANC follow-up, place of delivery, and mode of delivery. Residence was classified as urban and rural. Regions: Tanzania was divided into nine zones to assess geographical disparities in population indicators. The Ministry of Health’s Reproductive and Child Health Section employs this classification scheme, despite the fact that these zones are not formal administrative areas [19]. When regions are divided into zones, the denominators get larger and the sample errors for zonal indicators decrease. Mainland Tanzania was divided into eight administrative zones: Western zone: Tabora, Kigoma; Northern zone: Kilimanjaro, Tanga, and Arusha; Central zone: Dodoma, Singida, and Manyara. Southern Highlands zone: Iringa, Njombe, Ruvuma; Southern zone: Lindi, Mtwara; Southwest Highlands zone: Mbeya, Rukwa, Katavi, Songwe; Lake zone: Kagera, Mwanza, Geita, Mara, Simiyu, Shinyanga; Eastern zone: Dar es Salaam, Pwani, Morogoro. Zanzibar was grouped as a single zone (1 zone). Zanzibar Zone: Kaskazini Unguja, Kusini Unguja, Mjini Magharibi, Kaskazini Pemba, and Kusini Pemba [19]. Parity was defined as low if the mother gave birth once, multiparous if the mother had two to four births, and grand multiparous if the mother had five or more births [25]. ANC was evaluated to determine whether a woman who had a live birth within the previous two years received ANC from a skilled provider [20]. The number of ANC visits was divided into two categories: less than four visits and four or more [26].

### Data processing and analysis

This research was conducted using Demographic and Health Survey (DHS) data obtained from the official measure DHS website (https://dhsprogram.com/data/). The most recent live births of interviewed mothers in the 2 years preceding the survey (NR File) were examined to determine the dependent and independent variables. The DHS data in STATA format were cleaned, recoded, and appended to produce meaningful variables for study. STATA version 17 software was used to generate descriptive and analytic statistics.

To evaluate wealth-related inequalities in effective coverage of postnatal newborn care, the normalized concentration index and graph were used [27]. The concentration index (CIX) reflects wealth-related disparities in effective coverage of postnatal newborn care. The values range from −1 to 1, with a negative sign indicating effective coverage of postnatal newborn care concentration among the poor (pro-poor distribution) and a positive sign showing concentration among the wealthy (pro-rich distribution) [28]. However, because the outcome variable in this study is binary (effective postnatal newborn care provided or not), the concentration index’s bounds are set by its mean. To calculate the normalized concentration index, the Erreygers normalization correction was used (ECI) [27].


$$\begin{array}{c} \text{ECI}=\text{4}*\mu *\text{CI}(\text{y}) \end{array}$$ (1)

Where µ is the mean of effective coverage of postnatal newborn care and CI(y) is the generaliszed CI. A concentration curve is a graphical representation of the concentration index. To construct the concentration curve, the cumulative percentage of the study population was used, ranked by wealth, as the X-axis and the cumulative percentage of the study population, ranked by effective coverage of postnatal newborn care, as the Y-axis. A visual study of a concentration curve can reveal if it is above or below the line of equality. The concentration curve with a slope of 0 implies that there is no inequality; however, the concentration curves above and below the equality line (45 degrees) indicate that effective coverage of postnatal newborn care is disproportionately concentrated among the poor and rich, respectively [29].

Independent variables were used to decompose the overall wealth-related inequality in effective postnatal newborn care coverage. The breakdown’s outcome variable is wealth-related inequalities in effective coverage of postnatal newborn care. Sociodemographic and obstetric independent variables were all included in the decomposition analysis to determine their contribution to the inequality. The decomposition analysis was used to break down total wealth-related inequalities in effective coverage of postnatal newborn care into sociodemographic and obstetric determinants, as well as to explain how each possible determinant contributed to the observed discrepancy [28–31]. We used generalized linear models (GLM) to decompose the ECI and identify each variable’s impact on the effective coverage of postnatal newborn care coverage inequality. The effective coverage of postnatal newborn care model (y) can be expressed as follows:


$$\begin{array}{c} y=\mu +\sum_{k}\beta_{k}X_{k}+\in \end{array}$$ (2)

Where μ is an intercept, Xk is a set of variables that predict y, βk is the coefficient of Xk, and ∈ is the stochastic error term. The equation for decomposing ECI is as follows:


$$\begin{array}{c} ECI=\text{4}\left[\sum_{k=\text{1}}^{K}\beta_{k}{\overset{―}{X}}_{k}*CI+{GCI}_{\varepsilon }\right] \end{array}$$ (3)

In the equation, βk represents the partial effect of effective coverage of postnatal newborn care, CI is the concentration index of Xk, and GCI_∈_ is the generalized concentration index of the stochastic term of error. The equation suggests that a variable contributes to the inequality in the effective coverage of postnatal newborn care when it is correlated with effective coverage of postnatal newborn care’ prevalence and the variable is unequally distributed across the household wealth status. Variables with a higher partial effect and unequal distribution in relation to household wealth status contribute significantly to the inequality [28].

The overall ECI of effective postnatal care coverage includes both explained and unexplained components or residual. Elasticity refers to the change in the dependent variable (effective coverage of postnatal newborn care) when the independent variable changes by one unit. The concentration index for each variable reveals the extent and direction of socioeconomic inequality in healthcare access, as determined by certain independent variables. The absolute contribution is expressed in the same unit as the concentration index, while the percent contribution is the proportion of the concentration index that each variable makes to the overall observed wealth-related inequality in effective coverage of postnatal newborn care [28].

### Ethical consideration

Because our study was a secondary analysis of publicly available survey data from the MEASURE DHS program (https://dhsprogram.com/), no ethical approval or participant agreement was required. However, the Medical Research Council of Tanzania and the Zanzibar Health Research Institute have approved and authorized the 2022 TDHS-MIS questionnaire and survey protocol. The ICF’s Internal Review Board also examined the research. Following approval to use it, the data set was accessed on Sep 26, 2024 from the Demographic and Health Survey program. Furthermore, the dataset did not include any personally identifiable information, such as names or household numbers (identifiers).

## Results

### Socio‑demographic characteristics of respondents

This study included 4335 weighted sample women of reproductive age (15–49) who had given birth in the two years prior to the survey. The mean age of the respondents was 27.96 years (standard deviation: + 6.99 years), with more than three-fourths (70.02%) aged 20–34. More than half (55.29%) had primary education. 22.61% and 19.96% were classified as the poorest and poorer, respectively. Almost three-quarters of the study participants (72.48%) were from rural areas (Table 1), and one-third (33.92%) of the study respondents resided in the Lake administrative zone.

**Table 1:** Individual level socio-demographic and geographic characteristics of study participants for the study of Wealth-related inequalitie in effective coverage of postnatal newborn care in Tanzania.

Variable	Category	Weighted Frequency	Weighted percentage
Mothers’ age (in years)	15-19	428	9.88
	20-34	3036	70.02
	35-49	871	20.10
Sex of the household head	Male	3405	78.56
	Female	930	21.44
Marital status	Not married	1838	42.40
	Married	2497	57.60
Mothers’ education level	No education	894	20.63
	Primary	2397	55.29
	Secondary	993	22.90
	Higher	51	1.18
Work status	Working	2744	63.30
	Not working	1591	36.70
Wealth status	Poorest	980	22.61
	Poorer	865	19.96
	Middle	838	19.34
	Richer	851	19.62
	Richest	801	18.47
Residence	Urban	1193	27.52
	Rural	3142	72.48
Region	Western	445	10.28
	Northern	462	10.66
	Central	430	9.91
	Southern highlands	232	5.36
	Southern	174	4.01
	South West highlands	419	9.67
	Lake	1471	33.92
	Eastern	576	13.28
	Zanzibar	126	2.91

### Obstetrics realted characteristics of study participants

Among the respondents, more than half (52.20%) were multiparous, and nearly two-thirds (65.09%) of the respondents had four or more ANC visits. 18.84% delivered at home. Of all respondents, 89.33% had vaginal deliveries (Table 2).

**Table 2:** Obstetrics realted characteristics of study participants for the study of Wealth-related inequalitie in effective coverage of postnatal newborn care in Tanzania.

Variable	Category	Weighted Frequency	Weighted percentage
Parity	Low parity	987	22.76
	Multiparous	2263	52.20
	Grand multiparous	1085	25.04
ANC follow-up	Yes	3882	89.54
	No	453	10.46
Number of ANC visits	None	453	10.46
	Less than four visits	1060	24.45
	Four visits or more	2822	65.09
Place of delivery	Home	817	18.84
	Health facility	3518	81.16
Mode of delivery	Vaginal	3872	89.33
	Ceserian section	463	10.67

### Effective coverage of postnatal newborn care in Tanzania

**Fig 2:**
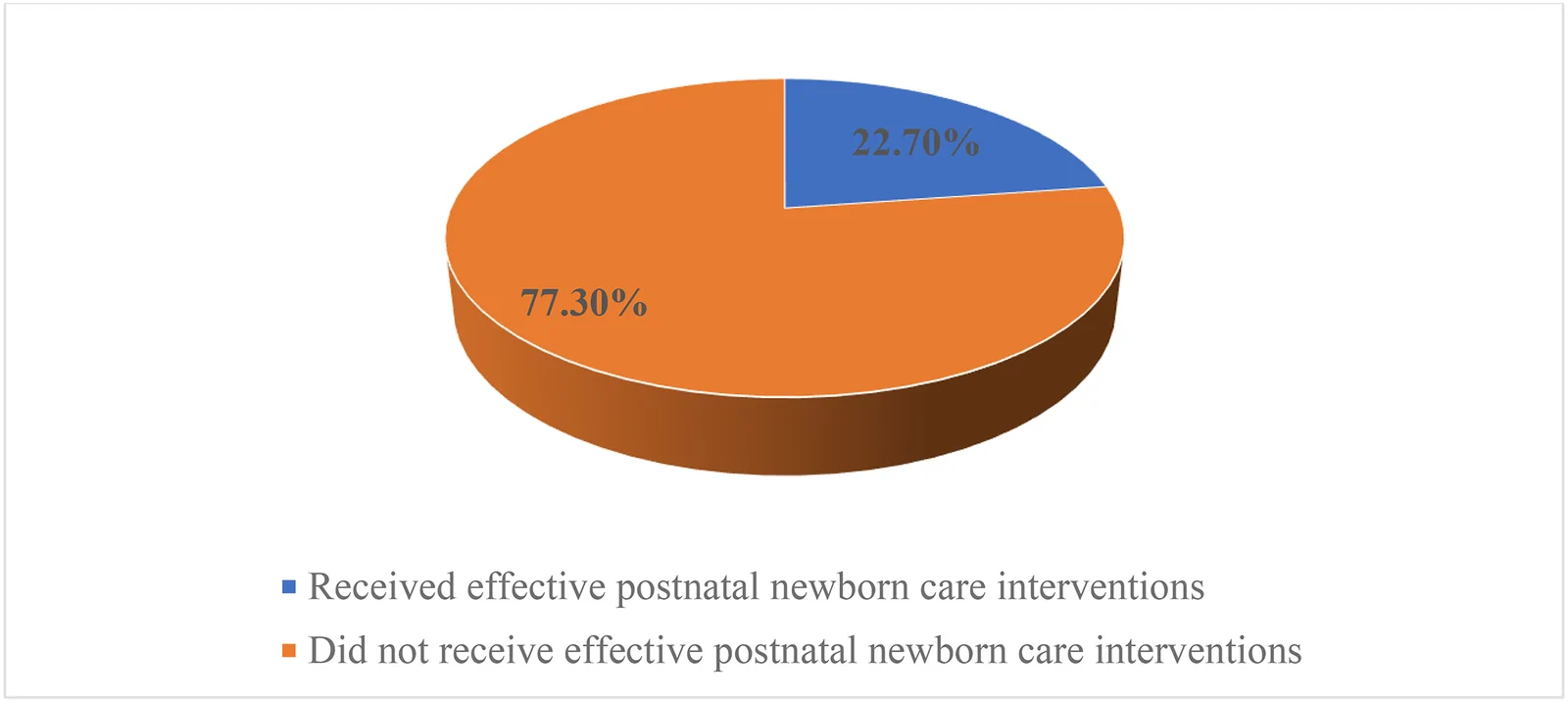
Proportion of newborns who received effective newborn care interventions in Tanzania.

**Fig 3:**
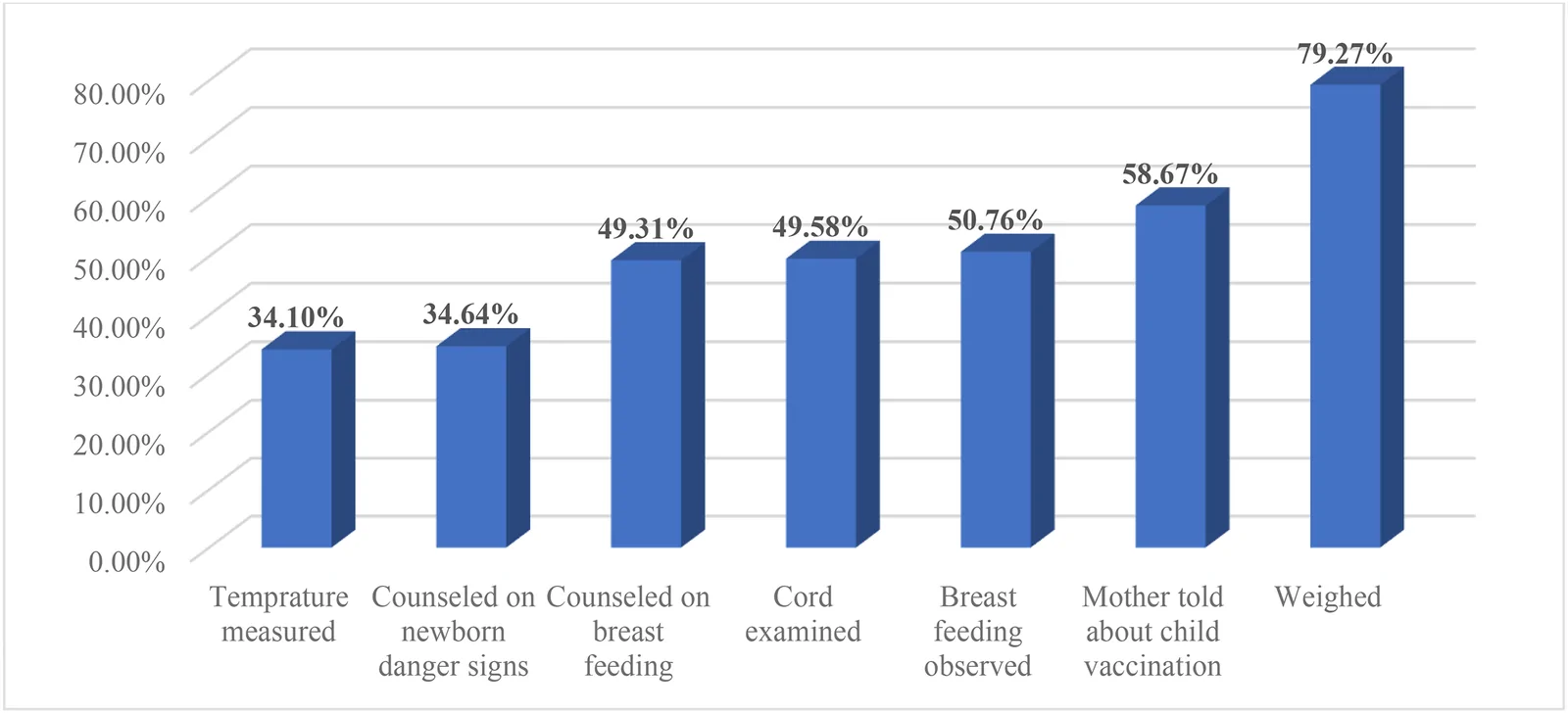
Proportion of newborns who received specific postnatal newborn care interventions in Tanzania.

The proportion of newborns who received effective postnatal newborn care interventions in Tanzania was 22.70% [95% CI: 21.48, 23.97] (Fig 2). The most common service that was provided was weighing, with 79.27% of newborns weighed at birth. While only 34.01% of newborns’ temperatures were measured in the first two days after birth (Fig 3). Furthermore, the proportions of effective newborn care provision were 29.14% and 22.9% among mothers with secondary and higher education, respectively. 7.29% of newborns with mothers who are grandmultiparous received effective newborn care, which is lower than the 10.83% reported in newborns with low parity mothers (Table 3). 17.14% of newborns from mothers who received four and greater than four ANC follow-ups received effective newborn care, while only 5.13% of newborns from mothers who received less than four ANC received effective newborn care. 17.82% of newborns delivered at health facilities received effective newborn care, whereas only 1.72% of newborns delivered at home received effective newborn care. 34% of newborns delivered through a cesarean section received effective newborn care compared to the 8.84% of newborns. delivered through vaginal delivery.

**Table 3:** Proportion of effective postnatal newborn care based on different characteristics in Tanzania.

Variable	Category	Effective postnatal newborn care		Total
		Yes (N) (%)	No (N) (%)	
Mothers’ age (in years)	15-19	101 (23.55)	327 (76.45)	428
	20-34	667 (21.96)	2369 (78.04)	3036
	35-49	217 (24.88)	655 (75.12)	871
Sex of the household head	Male	745 (21.90)	2660 (78.10)	3405
	Female	239 (25.65)	691 (74.35)	930
Marital status	Not married	440 (23.96)	1398 (76.04)	1838
	Married	544 (21.78)	1953 (78.22)	2497
Mothers’ education level	No education	103 (11.51)	791 (88.49)	894
	Primary	485 (20.23)	1912 (79.77)	2397
	Secondary	371 (37.34)	622 (62.66)	993
	Higher	25 (49.89)	26 (50.11)	51
Work status	Working	643 (23.43)	2101 (76.57)	2744
	Not working	341 (21.45)	1250 (78.55)	1591
Residence	Urban	464 (38.92)	729 (61.08)	1193
	Rural	520 (16.54)	2622 (83.46)	3142
Region	Western	42 (9.39)	403 (90.61)	445
	Northern	71 (15.41)	391 (89.46)	462
	Central	69 (16.09)	360 (83.91)	429
	Southern Highlands	141 (60.74)	92 (39.26)	233
	Southern	30 (17.18)	144 (82.82)	174
	South West Highlands	77 (18.48)	342 (81.52)	419
	Lake	299 (20.31)	1172 (79.69)	1471
	Eastern	229 (20.31)	347 (60.27)	576
	Zanzibar	26 (20.62)	100 (79.31)	126
Parity	Low parity	284 (28.76)	703 (71.24)	987
	Multiparous	536 (23.67)	1727 (76.33)	2263
	Grand multiparous	164 (15.17)	921 (84.83)	1085
ANC follow-up	Yes	919 (23.67)	2963 (76.33)	3882
	No	65 (14.37)	388 (85.63)	453
Number of ANC visits	None	65 (14.37)	388 (85.63)	453
	Less than four visits	178 (16.79)	882 (83.21)	1060
	Four visits or more	741 (26.26)	2081(73.74)	2822
Place of delivery	Home	31 (3.79)	786 (96.21)	817
	Health facility	953 (27.09)	2565 (72.91)	3518
Mode of delivery	Vaginal	744 (19.21)	3128 (80.79)	3872
	Ceserian section	241 (51.98)	222 (48.02)	463

### Wealth-related inequalities in effective newborn care coverage in Tanzania

**Fig 4:**
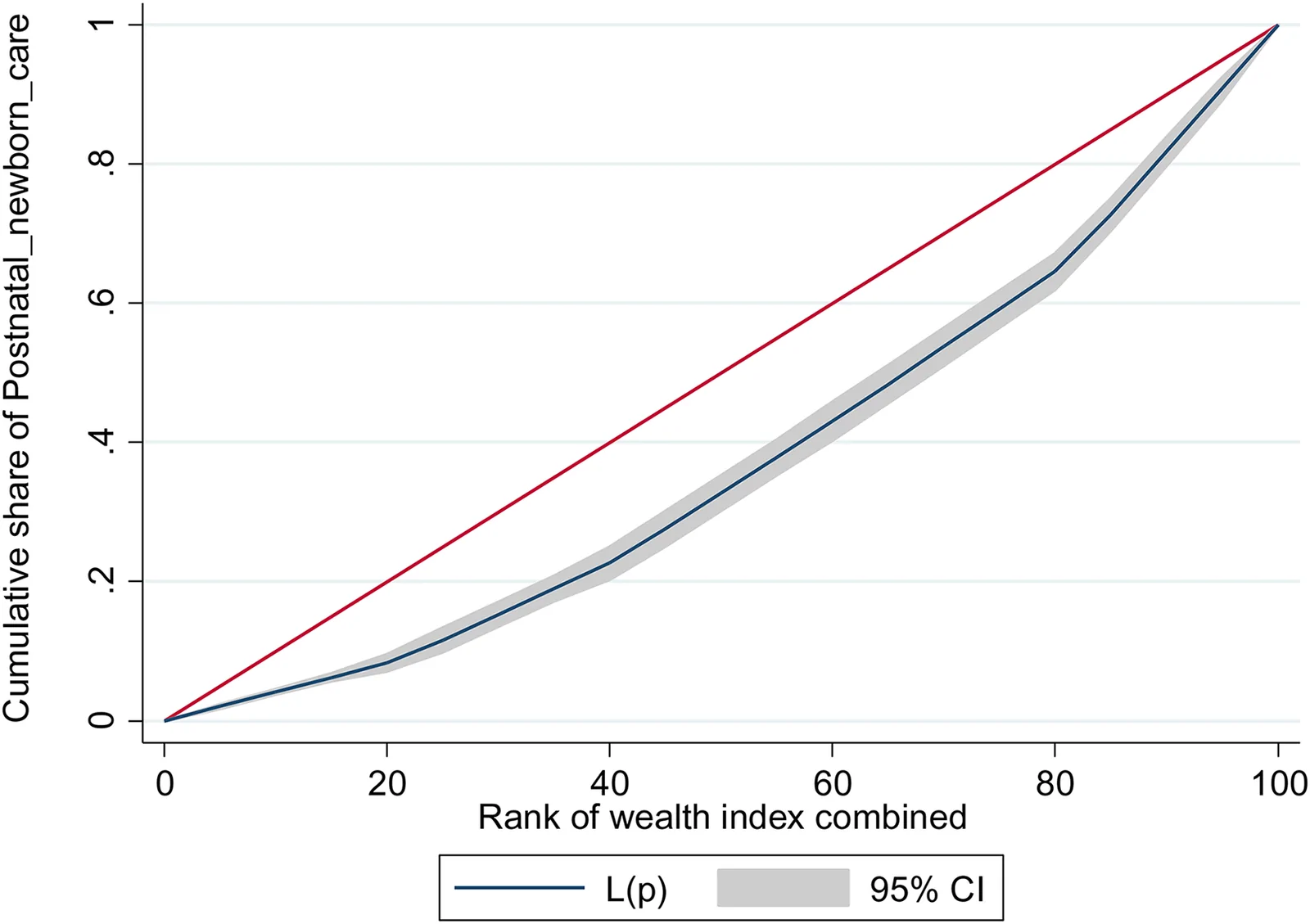
Wealth-related inequality in effective coverage of postnantal newborn care in Tanzania.

Our study found a pro-rich wealth-related inequality in effective postnatal newborn care coverage in Tanzania, with Erreygersconcentration index value of ECI = 0.26 (95% CI: 0.24, 0.29) (P < 0.001). This indicates that effective postnatal newborn care coverage is more concentrated among newborns from rich households. The concentration index findings are consistent with the concentration curve results. In Fig 4, the concentration curve for effective coverage of postnatal newborn care fell below the line of equality, indicating a more concentrated distribution in wealthy households (Fig 4).

### Decomposition of the wealth-related inequality in effective postnatal newborn care in Tanzania

In this study, the largest contributors to the observed wealth-related inequality were place of delivery (68.43), residence (22.72%), mothers’ education level (11.62%), region (6.84), ANC follow-up (4.49%), and mode of delivery (3.37%).

The elasticity finding revealed that health facility delivery had an elasticity of 0.684, indicating a positive association with effective newborn care coverage. However, since health facility deliveries were concentrated in rich households, it contributed positively to the pro-rich wealth-related inequality in effective newborn care coverage.

Moreover, newborns from urban areas contributed to 22.72% of wealth-related inequality in effective postnatal newborn care. Additionally, based on the elasticity finding, urban residence had an elasticity of 0.097, showing a positive association of urban residence and effective postnatal newborn care. This will simultaneously have a positive effect on the pro-rich wealth-related inequality in effective newborn care coverage as urban residence was more concentrated in rich households.

Furthermore, mothers’ education level explained 11.62% of the overall wealth-realted inequality in postnatal newborn care. Newborns delivered from mothers with secondary-level education contribute to 13.64% of the inequality. Secondary maternal education had an elasticity of 0.098, indicating a positive association with effective postnatal newborn care coverage. Because secondary education was concentrated among wealthier households, it contributed positively to the observed pro-rich inequality.

Regional variations contribute to 6.84% of the wealth-related inequality in effective postnatal newborn care, with Eastern, Lake, Southern Highlands, and South West Highlands explaining 5.42%, 1.67%, 0.57%, and −0.24% of the overall wealth-related inequality, respectively. Residence in the Eastern, Lake, and Southern Highlands regions had positive elasticities (0.084, 0.141, and 0.060, respectively), indicating positive associations with effective newborn care coverage. Because wealthier households were disproportionately represented among these regions, they contributed positively to the observed pro-rich inequality. Having an ANC follow-up described 4.49% of the wealth-related inequality in effective postnatal newborn care. ANC follow-up had an elasticity of 0.214, indicating a positive association with effective postnatal newborn care coverage. Since ANC follow-up was concentrated among wealthier households, it contributed positively to the observed pro-rich inequality.

Cesarean section delivery shared 3.37% of the total inequality in effective newborn care. Cesarean delivery had an elasticity of 0.066, indicating a positive association with effective newborn care coverage. (Table 4).

**Table 4:** Decomposition of the concentration index of wealth-related inequalities in effective coverage of postnatal newborn care in Tanzania.

Variable	Category	Coefficient (95% CI)	Elasticity	ECI	Contribution	Percent contribution (%)
Mothers’ education level	No education (Ref.)					
	Primary	0.021 (−0.009, 0.051)	0.080	−0.069	−0.006	−2.106
	Secondary	0.114 (0.076, 0.152) ***	0.098	0.365	0.036	13.644
	Higher	0.165 (0.055, 0.275) ***	0.006	0.034	<0.001	0.082
	Sub total				0.030	11.620
Residence	Urban	0.108 (0.079, 0.137) ***	0.097	0.615	0.060	22.716
	Rural (Ref.)					
Region	Western (Ref.)					
	Northern	0.024 (−0.026, 0.075)	0.013	−0.026	<−0.001	−0.133
	Central	0.037 (−0.014, 0.088)	0.019	−0.062	−0.001	−0.454
	Southern Highlands	0.399 (0.338, 0.461) ***	0.068	0.022	0.002	0.573
	Southern	0.014 (−0.053, 0.082)	0.006	−0.032	- < 0.001	−0.067
	South West Highlands	0.051 (−0.0002, 0.102) **	0.026	−0.024	−0.001	−0.243
	Lake	0.083 (0.042, 0.124) ***	0.141	0.031	0.004	1.672
	Eastern	0.171 (0.122, 0.221) ***	0.084	0.170	0.014	5.423
	Zanzibar	−0.001 (−0.078, 0.076)	0.003	0.051	<0.001	0.067
	Sub total				0.018	6.838
ANC follow-up	Yes	0.047 (0.010, 0.085) **	0.214	0.055	0.012	4.494
	No (Ref.)					
Place of delivery	Home (Ref.)					
	Health facility	0.127 (0.096, 0.157)***	0.684	0.263	0.180	68.426
Mode of delivery	Vaginal (Ref.)					
	Cesarean section	0.214 (0.175, 0.252) ***	0.066	0.134	0.009	3.374
	Total explained inequality				0.309	117.469
	Residual				−0.046	−17.469
	Overall inequality (ECI)				0.263	100
	95% confidence interval	ECI = 0.263 (0.236, 0.291)***				

ECI= Erreygers Concentration Index; **=P-value<0.05; ***=P-value<0.01; Ref.=Reference category.

## Discussion

The study looked at the extent and distribution of inequalities in effective coverage of postnatal newborn care in Tanzania. Addressing this issue is vital for implementing effective interventions to maximize the effective coverage of newborn care, improve child health, end preventable newborn deaths, and attain SDG 3. This study discovered that only 22.70% [95% CI: 21.48, 23.97] of newborns receive effective postnatal newborn care. Furthermore, effective coverage of postnatal newborn care is more concentrated in newborns from wealthy households, suggesting a pro-rich inequality (ECI = 0.26 (95% CI: 0.24, 0.29) (P < 0.001). The major contributors to the wealth-related inequality in effective coverage of postnatal newborn care were place of delivery, residence, mothers’ education level, region, ANC follow-up, and mode of delivery.

Our findings demonstrated a pro-rich wealth-related disparity in effective postnatal newborn care coverage. This finding is corroborated by a study conducted in Ethiopia utilizing 2016 data [15], which revealed that households with higher socioeconomic status were more likely to obtain effective postnatal newborn care. This is further supported by a study that examined the disparities in the provision of quality ANC in nine East African nations, revealing that the receipt of six ANC components was pro-rich within and across countries [32], including Tanzania. There is usually a substantial link between socioeconomic level and the usage of health care [15,33–36]. While maternal health services are offered free of charge in Tanzania, women may suffer indirect costs, such as transportation and opportunity costs [32], which frequently limits the utilization of institutional delivery and hence receiving postnatal newborn care, as the decomposition result showed place of delivery as a major contributor to the observed inequality.

The study’s findings found that place of delivery is the most significant factor contributing to wealth-related inequality in effective coverage of postnatal newborn care This finding is in line with studies conducted in Zambia [37], Uganda [38], Bangladesh [39], India [40] and evidence from national surveys in low- and middle-income countries [14], where institutional delivery was significantly associated with the utilization of postnatal care for the mother and newborn compared to home deliveries. This can be attributed to a combination of structural determinants, such as poor access to services, mothers’ perceived lack of services at health facilities, mothers’ perceived lack of importance in seeking postnatal newborn services, a shortage of community providers making routine home visits, costs and transportation difficulties, and other cultural, geographic, or financial barriers to seeking postnatal newborn care after home delivery [14,41]. This emphasizes the significance of increasing institutional delivery to ensure safe birth and newborn care, as well as focusing on quality beyond contact coverage, as contact coverage alone cannot ensure the provision of quality services and expected outcomes.

Residence is another contributor for the overall inequality, with wealth-related inequalities in effective coverage of postnatal newborn care observed among newborns from urban areas compared to newborns from mothers rural areas. This evidence is supported by studies that assessed postnatal newborn care in Nigeria [42] and SSA [23]. This can be due to the unequal distribution and location of healthcare facilities, women in urban areas have better healthcare access than those in rural areas. Furthermore, the fact that rural communities in SSA frequently lack access to healthcare due to inadequate infrastructure, financial constraints, poor road networks, and cultural norms that compel women to gain permission from their husbands can reduce effective newborn care among rural residents [43]. This highlights the importance of targeted interventions in rural areas, such as improving infrastructure and access to health care.

Mothers educational level showed an increase in the proportion of newborns from mothers who have secondary and higher education could result in increase in wealth-related inequality in effective postnatal newborn care, respectively. This evidence is supported by a systematic review and meta-analysis in Ethiopia [44], an analysis of maternal and newborn care in 20 countries in SSA [36], a study that included 21 SSA countries [23], and evidence from national surveys in low and middle-income countries [14]. Education contributes to increased awareness and knowledge of maternal and child health services, which favorably improves women’s desire/willingness to use them [32,45,46]. Furthermore, mothers benefit from education since they have better information about available and needed services and it gives them the ability to request them [47].

Effective postnatal newborn care was more concentrated in the Eastern, Lake, and Southern Highlands administrative zones than in the Western administrative zone. Regional disparities in gross domestic product (GDP) are one possible factor. Dar es Salaam, the capital city in the eastern administrative zone, has the highest GDP, while Mwanza, in the lake administrative zone, comes in second [48]. GDP has been linked to wealth and better healthcare access, resulting in higher provision and utilization of postnatal newborn care. This is evident since GDP has been related to child mortality, and a rise in GDP has been shown to improve child health and reduce child mortality [49,50]. Furthermore, the proportion of rich households is higher compared to other regions contributing to the association between wealth status and health service utilization. The proportion of rich households is higher in the above-mentioned administrative zones compared to other regions, contributing to the association between wealth status and health service utilization.

In our study, wealth-related inequalities in effective coverage of postnatal newborn care were evident among those whose mothers had ANC follow-up. This is also supported by a systematic review and meta-analysis of essential newborn care utilization in Ethiopia [44] and a study in SSA [23], which found a significant association between ANC follow-ups and newborn care. This could be due to ANC visits resulting in counseling about newborn practices among mothers during and after a pregnancy. This can be explained by the fact that mothers who received postnatal newborn care instruction throughout the ANC, birth, and PNC periods were more likely to grasp the need of postnatal newborn care [23]. Thus, the importance of ANC in utilizing effective postnatal newborn care should be emphasized, and increasing the proportion of mothers receiving ANC can increase postnatal newborn care utilization.

Cesarean section delivery was found to be a contributor to the wealth-related inequality in effective postnatal newborn care, with a rise in cesarean section delivered newborns raising the wealth-related inequality in the effective coverage of postnatal newborn care. This could arise from cesarean section deliveries occurring in health facilities. Previous studies conducted in Zambia [37], Bangladesh [39], and India [40] and this study have shown that institutional delivery is a predictor of postnatal newborn care utilization. As mothers who delivered at home might have poor access to services, a perceived lack of importance in seeking postnatal newborn services, a shortage of community health workers making routine home visits to home-delivered newborns, costs and transportation difficulties, and other cultural, geographic, or financial barriers to seeking postnatal newborn care after home birth [14,41].

### Limitations

Because the DHS does not directly measure income, this study used a wealth index as an indirect measure substitute for socioeconomic status. The study cannot establish a causal relationship between independent variables and effective coverage of postnatal newborn care, as it utilizes a cross-sectional study design. Additionally, the data was acquired through cross-sectional interviews with respondents’ self-reports; as a result, there is a possibility of social desirability and recall bias. This study relied on secondary data from respondents and did not consider service or health system-related factors that can impact effective coverage.

## Conclusion

In Tanzania, only one out of every five newborns has received effective postnatal newborn care, indicating a poor level of coverage, with postnatal newborn care disproportionately concentrated in wealthy households. Place of delivery, level of education, residency, region, ANC follow-up, and mode of delivery all had a substantial impact on the pro-rich disparities in effective postnatal newborn care coverage. Although facility delivery was associated with higher coverage, contact with health facilities did not consistently ensure receipt of all essential postnatal newborn care components. Policymakers and stakeholders should emphasize improving access to quality maternal and newborn services among disadvantaged populations, particularly those in rural areas, among home deliveries, and in poorer communities, while concurrent efforts should be made to improve economic status, maternal education, ANC utilization, and increase utilization of health facility delivery.

## Supporting information

S1 File STROBE checklist cross-sectional. (DOCX)

## Abbreviations

ANC: Antenatal Care
CI: Confidence Interval
CIX: Concetration Index
DHS: Demographic and Health Survey
EAs: Enumeration Areas
ECI: Erreygers Concentration Index
GDP: Gross Domestic Product
NMR: Neonatal Mortality Rate
SDGs: Sustainable Development Goals
SSA: Sub-Saharan Africa
TDHS-MIS: Tanzania Demographic and Health Survey-Malaria Indicator Survey
WHO: World Health Organization
