# Supplementary material for: Wealth-related inequality in effective coverage of postnatal newborn care in Tanzania; A decomposition analysis of the Tanzania Demographic and Health Survey, 2022
Source: PLoS One. 2026 Sep 17;21(9):e0358220. doi: 10.1371/journal.pone.0358220 (PMC13585273; doi:10.1371/journal.pone.0358220)
Supplement: Supplementary file 2 [file pone.0358220.s002.docx]

Wealth-related inequality in effective coverage of postnatal newborn care in Tanzania; A decomposition analysis of the 2022 Tanzania Demographic and Health Survey.

1. Conclusion: the study found a low level of effective postnatal newborn care coverage, with a pro-rich distribution. Focus should be given on increasing institutional deliveries, expanding education, promoting ANC follow-up, improving wealth status, and newborns from vaginal deliveries. Furthermore, authorities and stakeholders should work on expanding health services in rural and affected regions to increase the population's access to newborn health services. (42-46)

The conclusion accurately identifies key determinants (e.g., wealth status, rural access) but remains vague on the 'how.' To strengthen the impact and utility of the findings for policymakers and stakeholders, the authors should provide more specific, actionable recommendations.

2. The postnatal newborn care includes: thermal protection (e.g., promoting skin-to-skin contact between mother and infant); hygienic umbilical cord and skin care; early and exclusive breastfeeding; screening for signs of serious health problems or need for additional care (e.g., low birth weight, sick, or Human immunodeficiency virus infected mother); and preventive treatment (e.g., Bacillus Calmette-Guérin vaccine (BCG) and Hepatitis B immunizations, vitamin K, and ocular prophylaxis). (93-98)

The summary of postnatal care practices is accurate; however, it currently lacks citations. To strengthen the manuscript and allow readers to verify the guidelines, please add references from established clinical guidelines (e.g., WHO, UNICEF, or relevant national bodies) for each of the points mentioned.

3. Most studies use crude coverage indicators, which are frequently used in maternal and child health studies, representing the tip of the iceberg because they only consider contact between women or children and health care providers. They are unable to show the level of adherence to care standards, the manner in which services are delivered, or the quality of care offered. (117-120)

The authors correctly identify the limitation of 'crude coverage' indicators. However, the manuscript must explicitly state how their own study's methodology addresses this gap. Please specify: What specific components of your 'effective coverage' metric directly measure quality of care, adherence to guidelines, or the process of service delivery, thereby moving beyond simple contact with a provider?

4. The introduction does not clearly define a concrete research gap. Please explicitly state the specific knowledge void that your study aims to fill.
5. This study included 4335 weighted sample women of reproductive age (15–49) who had given birth in the two years preceding the survey.

Please clarify the sampling procedure. How were the 4,335 participants selected from the larger survey? What were the specific eligibility criteria (inclusion/exclusion) for this particular analysis?

6. The authors state that the Erreygers normalized concentration index was used, and while the concentration curve graph is helpful for visualization, the precise numerical values are missing. For the sake of transparency, reproducibility, and to allow readers to ascertain the exact magnitude of inequality, please provide a table of the calculated Erreygers index values, either in the main manuscript or as a supplementary appendix.
7. All figures in the manuscript are currently missing a caption. This is a critical omission that must be corrected.
8. Authors have mentioned “Possible explanations for this significant variation could be the later study used a video-recorded observation, while this study used respondents’ responses only.” Please elaborate on this statement. How is this statement providing any justification?
9. The limitations section should acknowledge that the use of cross-sectional data prevents the establishment of causal relationships between the identified factors (e.g., wealth, education) and the outcome of effective postnatal care. Please add this standard but critical limitation.
10. The manuscript lacks a dedicated section on policy recommendations. Based on your key findings, specifically the pro-rich inequality and the determinants of effective care, please add clear, actionable recommendations for policymakers and stakeholders.
