# Supplementary material for: Wealth-related inequality in effective coverage of postnatal newborn care in Tanzania; A decomposition analysis of the Tanzania Demographic and Health Survey, 2022
Source: PLoS One. 2026 Sep 17;21(9):e0358220. doi: 10.1371/journal.pone.0358220 (PMC13585273; doi:10.1371/journal.pone.0358220)
Supplement: Supplementary file 3 [file pone.0358220.s003.docx]

**Response letter**

**Journal: PLOS One**
Title: **Wealth-related inequality in effective coverage of postnatal newborn care in Tanzania; A decomposition analysis of the 2022 Tanzania Demographic and Health Survey.**

**Submission ID:  PONE-D-25-19119
Corresponding Author:** Amanuel Yosef
Dear Pritam Ghosh, Ph. D. (Editor) and Reviewers,
We would like to forward our deepest gratitude for the constructive, helpful and insightful comments given to this manuscript by the editor and reviewers which are highly helpful in improving the substance and contents of our manuscript. We considered each comment and clarify questions and comments raised by the reviewers on the manuscript thoroughly. Our point by-point responses for each comment and questions are described in detailed on the following pages. Further, the details of changes were shown by track changes in the supplementary document attached. Thank you. N.B. The page numbers and line numbers mentioned are for the revised manuscript with tracked changes.

Sincerely

Amanuel Yosef (corresponding author)

| S.N | Editor/Reviewer comments/questions | Responses |
| --- | --- | --- |
| **Reviewers’ comments** | | |
| **Reviewer 1** | | |
| 1. | The conclusion accurately identifies key determinants (e.g., wealth status, rural access) but remains vague on the 'how.' To strengthen the impact and utility of the findings for policymakers and stakeholders, the authors should provide more specific, actionable recommendations. | Dear reviewer, we appreciate the time and effort given to reviewing our paper. The provided comments have enabled us to improve the manuscript's content. Thank you.  We have amended the conclusion section and provided specific policy recommendations in the conclusion; however, we were unable to include those detailed recommendations in the abstract section due to the word limit to the abstract section. |
| 2. | The summary of postnatal care practices is accurate; however, it currently lacks citations. To strengthen the manuscript and allow readers to verify the guidelines, please add references from established clinical guidelines (e.g., WHO, UNICEF, or relevant national bodies) for each of the points mentioned. | Dear reviewer, thank you for the comment. The postnatal recommendations are taken from two WHO references, and we have included the citations. PN 5, LN 98. |
| 3 | The authors correctly identify the limitation of 'crude coverage' indicators. However, the manuscript must explicitly state how their own study's methodology addresses this gap. Please specify: What specific components of your 'effective coverage' metric directly measure quality of care, adherence to guidelines, or the process of service delivery, thereby moving beyond simple contact with a provider? | Dear reviewer, Considering your comment, we have described that our study assessed whether or not the contact between newborns and healthcare providers resulted in the provision of standard newborn care services, focusing on the process aspect of healthcare quality leaving out structural and outcome components of quality, as the data set doesn't include variables that can assess those components. Thank you. PN 6, LN 123-125. |
| 4 | The introduction does not clearly define a concrete research gap. Please explicitly state the specific knowledge void that your study aims to fill. | Dear reviewer, thank you for the comment. We have tried to clearly state the research gap by linking the evidence gap with the Tanzania Health Sector Strategic Plan V. Please refer to PN 6, LNs 116-119 and PNs 6 & 7 and LNs 126-137. |
| 5 | Please clarify the sampling procedure. How were the 4,335 participants selected from the larger survey? What were the specific eligibility criteria (inclusion/exclusion) for this particular analysis? | Dear reviewer, we have included a sampling procedure figure. Thank you. Please refer to figure 1. We have also described the inclusion and exclusion criteria. PN 8, LNs 167-169. |
| 6 | The authors state that the Erreygers normalized concentration index was used, and while the concentration curve graph is helpful for visualization, the precise numerical values are missing. For the sake of transparency, reproducibility, and to allow readers to ascertain the exact magnitude of inequality, please provide a table of the calculated Erreygers index values, either in the main manuscript or as a supplementary appendix. | Dear reviewer, thank you for the concern. The numerical value of the Erreygers concentration index was already included in the main text of the manuscript and at the end of table 4 with the 95% confidence interval and the p-value. Please refer to PN 21, LNs 326-327. |
| 7. | All figures in the manuscript are currently missing a caption. This is a critical omission that must be corrected. | Dear reviewer, apologies for the confusion. PLOS One's submission guideline requires figure captions to be inserted in the main text immediately following the text that describes each figure, and we have already included figure captions in the main manuscript document. |
| 8 | Authors have mentioned “Possible explanations for this significant variation could be the later study used a video-recorded observation, while this study used respondents’ responses only.” Please elaborate on this statement. How is this statement providing any justification? | Dear reviewer, Video-recorded observations by experts tend to estimate the provision of services more accurately compared to responses from study participants. The following reasons can be considered: 1. Respondents may not fully understand or differentiate the services they received or not. 2. The deliveries occur within a period of two years prior to the study, which may introduce recall bias. Due to those factors, our study may inflate the provision of effective postnatal newborn care compared to the study that used video-recorded observation. Furthermore, other justifications are also mentioned, such as the use of stronger criteria. Thank you. Elaborations are included in PN 30 & 31, LNs 405-407. |
| 9 | The limitations section should acknowledge that the use of cross-sectional data prevents the establishment of causal relationships between the identified factors (e.g., wealth, education) and the outcome of effective postnatal care. Please add this standard but critical limitation. | Dear reviewer, thank you for the suggestion. We have included the specific limitation. PN 35, LNs 513-515. |
| 10 | The manuscript lacks a dedicated section on policy recommendations. Based on your key findings, specifically the pro-rich inequality and the determinants of effective care, please add clear, actionable recommendations for policymakers and stakeholders. | Dear reviewer, we appreciate the important suggestion. We have included policy recommendations based on the findings. Since PLOS One doesn't indicate to include a specific policy recommendation section, we have included our policy recommendation in the conclusion section. Please refer to the conclusion section. |
| Reviewer 2 | | |
| 1 | Firstly, I would like to commend the authors on their well-structured and scientifically rigorous study. I have identified a few areas where the manuscript could be improved to enhance its clarity and impact. Please find my specific comments below | Dear reviewer, we appreciate the time and effort in reviewing our paper, as well as your helpful recommendations. It has enabled us to improve the manuscript's content. Thank you. |
| 2 | The introduction lacks a clear structure and logical flow. The research gaps and research questions are unclear. I suggest the authors to set a context by explaining why studying wealth-based inequality in effective coverage of postnatal newborn care is important in Tanzania. | Dear Reviewer, Thank you for your comment. We tried to clarify the research gap by relating it to Tanzania's Health Sector Strategic Plan V. Please see PN 6, LNs 116-119, PNs 6 and 7, and LNs 126-137. |
| 3 | It is better to rewrite based a) what the issue is, b) why it is important, c) what the literature says, d) what the research gap (Theoretical knowledge, empirical, evidence, population, methodological gaps, etc.) and, e) what the question in this study are, f) objectives of the study or how this study could contribute to the literature. | Dear reviewer, Thank you for your valuable input. We have carefully considered your suggestions and have made revisions to the introduction section. We have started by introducing the Sustainable Development Goals (SDGs) in relation to the target of reducing neonatal mortality. We have also included information on why the neonatal period is a critical public health concern, including global, regional, and country-specific data (such as from Tanzania). Additionally, we have highlighted the importance of effective postnatal newborn care in reducing neonatal mortality and morbidity and have included literature on coverage rates in different countries. Finally, we have outlined the research gaps, research questions, and study aims in the last paragraphs. Please refer to the introduction section. |
| 4 | Yet, to be of interest for readers outside Tanzania, too, the paper should also refer to some studies in the international literature, to highlight the gaps and unanswered questions, the methodologies used, etc. This could also help with the interpretation of the results, as it could allow international comparisons. | Dear reviewer, we appreciate the important suggestion. Based on your feedback, we have tried to spotlight the gaps and unanswered questions of previous studies in the background section. Please see PN 7 LNs 134-139. Further detailed methodological variations and gaps in the discussion section. Thank you. |
| 5 | The authors have tried to explained Wealth-related inequality in effective coverage of postnatal newborn care. Here household’s wealth is the exposure variables. The authors are trying to estimate factors contributing to the existing inequality across wealth class. How wealth categories can be taken as a factor in this decomposition model (Check table 3 and 4)? | Dear reviewer, Thank you for bringing up an important question. You are absolutely correct in stating that wealth should not be considered as an explanatory variable. We initially included wealth as an explanatory variable because when we excluded it, the sum of the percent contribution of the remaining explanatory variables exceeded 100%. Additionally, some articles have included wealth as an explanatory variable in their decomposition analysis. However, we have since updated our analysis and have excluded the wealth index as an explanatory variable. This is because it is common for the percentage contribution of a regression-based decomposition analysis of wealth-related inequality to be greater than 100%. Please refer to tables 3 and 4 for the updated results. Thank you for your valuable feedback. |
| 6 | In some part, the authors repeated the results. It would be more beneficial if the discussion primarily emphasizes the key findings, their interpretations, and the implications they carry for public health interventions and potential policy adjustments. For improved coherence and academic rigor, the discussion section may be organized into the following three sub-sections: Key Findings, Policy Recommendations, Strengths and Limitations. These additions would greatly enhance the overall quality and policy relevance of the manuscript. | Dear reviewer, based on your suggestion, we have removed some repetitive statements from the discussion section that show results already mentioned in the result section. The key findings are already summarized in the first paragraph, whereas some policy recommendations or implications are mentioned in the paragraphs. Furthermore, specific policy recommendations are included and updated in the conclusion section inline with your and reviewer one's recommendation. Please refer to the discussion, limitation and recommendation sections. Thank you. |
| 7 | Authors can also compare the results of different methods and assess which was more fruitful and why. Discuss the implications of this research and compare it to the results of other studies. Authors can cite related studies to show how your study compares (in addition to the introduction). | Dear reviewer, Thank you for your valuable feedback. We have carefully reviewed the different methods used in our study and compared them to those used in other studies. Through this comparison, we have highlighted the strengths and weaknesses of our study in relation to the others. These methodological variations include differences in data collection techniques, criteria, measurement techniques, and study scales. For specific examples, please refer to PN 28, LNs 427-431; PN 29, LNs 431-432; PN 29, LNs 435-437; PN 29, LNs 439-442; PN 29, LNs 451-454. |
| 8 | One paragraph on the policy recommendation. In this paragraph, the authors must highlight, how the findings of this study help the policy makers to develop new policies to improve the current situation and to solve this problem based on this current study findings, or how the study findings will help the policymakers to modify existing policies in this field. | Dear reviewer, to address the comments of both reviewers, we have updated and included detailed policy recommendations in the conclusion section. We believe that adding a separate paragraph for policy recommendations in the discussion section would make the conclusion section redundant. Please refer to the conclusion section. Thank you. |
