# Supplementary material for: Wealth-related inequality in effective coverage of postnatal newborn care in Tanzania; A decomposition analysis of the Tanzania Demographic and Health Survey, 2022
Source: PLoS One. 2026 Sep 17;21(9):e0358220. doi: 10.1371/journal.pone.0358220 (PMC13585273; doi:10.1371/journal.pone.0358220)
Supplement: Supplementary file 4 [file pone.0358220.s004.docx]

**Title: Wealth-related inequality in effective coverage of postnatal newborn care in Tanzania; A decomposition analysis of the 2022 Tanzania Demographic and Health Survey.**

Thank you for entrusting me with the opportunity to review the manuscript for your esteemed journal. After conducting a thorough and critical review, I would like to highlight several findings that require attention. I recommend accepting this article once the necessary corrections have been implemented.

Minor comments:

1. Revised the title as “Wealth-related inequality in effective coverage of postnatal newborn care in Tanzania: A decomposition analysis of the Tanzania Demographic and Health Survey”
2. Methods section of the abstract need to be revised as structural issues exists.
3. Line 318 “**Obstetrics realted characteristics of study participants**” revised with “**Obstetrics related characteristics of study participants**” and title of the Table 2.
4. Include ECI value in the table 4 or in separate table, with p-vale, unexplained/error.
5. It would be good if a inequality curve present based on wealth-based ECI value to reflects the inequality inequalities in effective newborn care coverage (revise).
6. Discussion needs to be revised which should not repeated the results only rather it should be mentioned the possible reasons and the factors of the outcomes. Citing only the research article also not be appropriate always, it should be discussed the results with the previous literatures.
7. Conclusion is too generic, and overstated; conclusion need to be condensed.
