# Supplementary material for: Wealth-related inequality in effective coverage of postnatal newborn care in Tanzania; A decomposition analysis of the Tanzania Demographic and Health Survey, 2022
Source: PLoS One. 2026 Sep 17;21(9):e0358220. doi: 10.1371/journal.pone.0358220 (PMC13585273; doi:10.1371/journal.pone.0358220)
Supplement: Supplementary file 5 [file pone.0358220.s005.docx]

**Response letter**

**Journal: PLOS One**
Title: **Wealth-related inequality in effective coverage of postnatal newborn care in Tanzania; A decomposition analysis of the 2022 Tanzania Demographic and Health Survey.**

**Submission ID:  PONE-D-25-19119R1
Corresponding Author:** Amanuel Yosef
Dear Pritam Ghosh, Ph. D. (Editor) and Reviewers,
We would like to forward our deepest gratitude for the constructive, helpful and insightful comments given to this manuscript by the editor and reviewers which are vital in improving the substance and contents of our manuscript. We considered each comment and clarify questions and comments raised by the reviewers on the manuscript thoroughly. Our point by-point responses for each comment and questions are described in detailed on the following pages. Further, the details of changes were shown by track changes in the supplementary document attached. Thank you. N.B. The page numbers and line numbers mentioned are for the revised manuscript with tracked changes.

Sincerely

Amanuel Yosef (corresponding author)

| S.N | Editor/Reviewer comments/questions | Responses |
| --- | --- | --- |
| **Editor’s comment** | | |
| 1. | The authors have made commendable efforts to upgrade the manuscript. I have identified a few areas where the manuscript could be improved to enhance its clarity and impact. Please find my specific comments below: | Dear Editor, we appreciate the time and effort given to reviewing our paper. The provided comments have enabled us to improve the manuscript's content. Thank you. |
| 2. | The research gaps and research questions are unclear. The Introduction section should be reorganised to follow a more standard academic structure: (a) a clear presentation of the context and background of the study; (b) a focused literature review that synthesises existing evidence, identifies research gaps (Theoretical, Knowledge, empirical, evidence, population, methodological gap etc.), and culminates in clearly articulated research questions; and (c) a concluding paragraph that explicitly outlines the research objectives. | Dear Editor, Thank you for the feedback. The comments provided by you are related to what has been provided by reviewer two in the previous revision. Paragraph one provided the context and background of the study,  while paragraphs 2-3 showed what existing literature and evidence show on the specific matter. Whereas paragraph 4 stated the research gaps and research questions 1 to 3. Finally, the last paragraph included a "therefore" statement to clearly describe the objectives/aims of the study. Please refer to the introduction section. |
| 3 | Discussion section must be improved dramatically. In some part the authors repeated the results. It would be more beneficial if the discussion primarily emphasized the key findings, their interpretations, and the implications they carry for public health interventions and potential policy adjustments. For improved coherence and academic rigor, the discussion section may be organized into the following three sub-sections: Key Findings, Policy Recommendations, Limitations and Strengths. These additions would greatly enhance the overall quality and policy relevance of the manuscript. | Dear Editor, based on reviewer two's previous feedback, we have removed some repetitive statements from the discussion section that show results already mentioned in the result section. We have also removed a section that compares the prevalence reported in this study with other studies to shorten the discussion section. The key findings are already summarized in the first paragraph, whereas some policy recommendations or implications are mentioned in the paragraphs. Furthermore, specific policy recommendations are included and updated in the conclusion section inline with you, reviewer three and previously reviewer two's recommendations. Please refer to the discussion, limitation, and recommendation sections. Thank you. |
| 4 | Each major finding should be discussed in a separate paragraph. Since the study likely addresses multiple research questions and objectives, devote one paragraph to each key finding. In these paragraphs, the authors should justify the results with reference to previous studies— (a) highlighting both similarities and differences. (b) Additionally, explain why and how the observed findings emerged in this study. | Dear Editor, Thank you for the valuable insight. We structured and updated the discussion section by providing separate paragraphs for each major finding. Please refer to the discussion section. |
| Reviewer 3 | | |
| 1 | Thank you for entrusting me with the opportunity to review the manuscript for your esteemed journal. After conducting a thorough and critical review, I would like to highlight several findings that require attention. I recommend accepting this article once the necessary corrections have been implemented | Dear reviewer, we value the time and effort you have invested in evaluating our paper, along with your constructive suggestions. We appreciate it. Thank you. |
| 2 | Revised the title as “Wealth-related inequality in effective coverage of postnatal newborn care in Tanzania: A decomposition analysis of the Tanzania Demographic and Health Survey” | Dear reviewer, Thank you for your comment. We have revised the title based on your suggestion. Please refer to the title. |
| 3 | Methods section of the abstract need to be revised as structural issues exists. | Dear reviewer, Thank you for the feedback. We have revised the method section of the abstract. Please refer to PN 3, LNs 48-55. |
| 4 | Line 318 “**Obstetrics realted characteristics of study participants**” revised with “**Obstetrics related characteristics of study participants**” and title of the Table 2. | Dear Reviewer, Thank you for your feedback. Based on our understanding, you are suggesting that we remove the text between the specific section title and Table 2. However, we believe that it is important to provide a brief description of the table's contents before presenting it, so that readers can easily understand the summary of results in the table |
| 5 | Include ECI value in the table 4 or in separate table, with p-vale, unexplained/error. | Dear Reviewer, Thank you for your comment. We have included the ECI with a 95% confidence interval and have also provided both the explained and unexplained values. Additionally, we have indicated the p-value for the ECI with asterisks to show that it is less than 0.001. Please refer to table 4. |
| 6 | It would be good if a inequality curve present based on wealth-based ECI value to reflects the inequality inequalities in effective newborn care coverage (revise). | Dear reviewer, a concentration curve was already provided and uploaded. Please refer to Figure 4. Thank you. |
| 7 | Discussion needs to be revised which should not repeated the results only rather it should be mentioned the possible reasons and the factors of the outcomes. Citing only the research article also not be appropriate always, it should be discussed the results with the previous  literatures. | Dear reviewer, Thank you for your valuable feedback. We also received similar feedback from reviewer one in the first revision and have made significant changes accordingly. In each paragraph, we have supported our results by comparing them with previous publications, and we have also included potential justifications for the observed results. Furthermore, we have removed any repeated results to avoid redundancy. However, as our study focuses on assessing prevalence, wealth-related inequality, and decomposition of factors, the discussion section may have become too broad. In order to incorporate your feedback and make the discussion section more concise and focused, we have removed the section that compares the prevalence reported in our study with that of other studies. Please refer to the discussion section. |
| 8 | Conclusion is too generic, and overstated; conclusion need to be condensed. | Dear reviewer, thank you for your valuable feedback. We have revised and condensed the conclusion section to focus solely on the findings presented in the study. Please refer to the updated conclusion section. |
