# Supplementary material for: Wealth-related inequality in effective coverage of postnatal newborn care in Tanzania; A decomposition analysis of the Tanzania Demographic and Health Survey, 2022
Source: PLoS One. 2026 Sep 17;21(9):e0358220. doi: 10.1371/journal.pone.0358220 (PMC13585273; doi:10.1371/journal.pone.0358220)
Supplement: Supplementary file 6 [file pone.0358220.s006.docx]

**Response letter**

**Journal: PLOS One**
Title: **Wealth-related inequality in effective coverage of postnatal newborn care in Tanzania; A decomposition analysis of the Tanzania Demographic and Health Survey, 2022.**

**Submission ID:  PONE-D-25-19119R2
Corresponding Author:** Amanuel Yosef Gebrekidan
Dear Pritam Ghosh, Ph. D. (Editor),

We would like to forward our deepest gratitude for the constructive, beneficial, and insightful comment given to this manuscript by the editor, which was vital in improving the substance and contents of our manuscript. We considered the editor’s comment on the manuscript thoroughly. The detail of the change is shown by track changes in the supplementary document attached. Thank you.

Sincerely,

Amanuel Yosef Gebrekidan

| S.N | Editor/Reviewer comments/questions | Responses |
| --- | --- | --- |
| **Editor’s comment** | | |
| 1. | One minor correction is required. You have to chnage the title slightly.  Insearfd of following title:  "Wealth-related inequality in effective coverage of postnatal newborn care in Tanzania; A decomposition analysis of the Tanzania Demographic and Health Survey."  The authors need to add the year of the Tanzania Demographic and Health Survey.  I think it will be like following:  "Wealth-related inequality in effective coverage of postnatal newborn care in Tanzania; A decomposition analysis of the Tanzania Demographic and Health Survey, 2022" | Dear Editor, we appreciate the time and effort given to reviewing our paper. Based on your suggestion, we have amended the title. Please refer to the title of the manuscript. Thank you. |
