# Supplementary material for: Wealth-related inequality in effective coverage of postnatal newborn care in Tanzania; A decomposition analysis of the Tanzania Demographic and Health Survey, 2022
Source: PLoS One. 2026 Sep 17;21(9):e0358220. doi: 10.1371/journal.pone.0358220 (PMC13585273; doi:10.1371/journal.pone.0358220)
Supplement: Peer review history

# Peer review: Decision Letter 0

**DOI:** 10.1371/journal.pone.0358220.r001

**Authors:** Pritam Ghosh

28 Oct 2025

Dear Dr. Gebrekidan,

Thank you for submitting your manuscript to PLOS ONE. After careful consideration, we feel that it has merit but does not fully meet PLOS ONE’s publication criteria as it currently stands. Therefore, we invite you to submit a revised version of the manuscript that addresses the points raised during the review process.

Please submit your revised manuscript by Dec 12 2025 11:59PM. If you will need more time than this to complete your revisions, please reply to this message or contact the journal office at plosone@plos.org. When you're ready to submit your revision, log on to https://www.editorialmanager.com/pone/ and select the 'Submissions Needing Revision' folder to locate your manuscript file.

- A rebuttal letter that responds to each point raised by the academic editor and reviewer(s). You should upload this letter as a separate file labeled 'Response to Reviewers'.
- A marked-up copy of your manuscript that highlights changes made to the original version. You should upload this as a separate file labeled 'Revised Manuscript with Track Changes'.
- An unmarked version of your revised paper without tracked changes. You should upload this as a separate file labeled 'Manuscript'.

If you would like to make changes to your financial disclosure, please include your updated statement in your cover letter. Guidelines for resubmitting your figure files are available below the reviewer comments at the end of this letter.

If applicable, we recommend that you deposit your laboratory protocols in protocols.io to enhance the reproducibility of your results. Protocols.io assigns your protocol its own identifier (DOI) so that it can be cited independently in the future. For instructions see: https://journals.plos.org/plosone/s/submission-guidelines#loc-laboratory-protocols. Additionally, PLOS ONE offers an option for publishing peer-reviewed Lab Protocol articles, which describe protocols hosted on protocols.io. Read more information on sharing protocols at https://plos.org/protocols?utm_medium=editorial-email&utm_source=authorletters&utm_campaign=protocols.

We look forward to receiving your revised manuscript.

Kind regards,

Pritam Ghosh, Ph. D.

Academic Editor

PLOS ONE

**Journal Requirements:**

When submitting your revision, we need you to address these additional requirements.

1. Please ensure that your manuscript meets PLOS ONE's style requirements, including those for file naming. The PLOS ONE style templates can be found at

https://journals.plos.org/plosone/s/file?id=wjVg/PLOSOne_formatting_sample_main_body.pdf and

https://journals.plos.org/plosone/s/file?id=ba62/PLOSOne_formatting_sample_title_authors_affiliations.pdf

2. Your ethics statement should only appear in the Methods section of your manuscript. If your ethics statement is written in any section besides the Methods, please delete it from any other section.

3. If the reviewer comments include a recommendation to cite specific previously published works, please review and evaluate these publications to determine whether they are relevant and should be cited. There is no requirement to cite these works unless the editor has indicated otherwise.

[Note: HTML markup is below. Please do not edit.]

Reviewers' comments:

Reviewer's Responses to Questions

**Comments to the Author**

1. Is the manuscript technically sound, and do the data support the conclusions?

Reviewer #1: Yes

Reviewer #2: Partly

**********

2. Has the statistical analysis been performed appropriately and rigorously?

Reviewer #1: Yes

Reviewer #2: Yes

**********

3. Have the authors made all data underlying the findings in their manuscript fully available?

The PLOS Data policy

Reviewer #1: Yes

Reviewer #2: Yes

**********

4. Is the manuscript presented in an intelligible fashion and written in standard English?

Reviewer #1: Yes

Reviewer #2: Yes

**********

Reviewer #1: Wealth-related inequality in effective coverage of postnatal newborn care in Tanzania; A decomposition analysis of the 2022 Tanzania Demographic and Health Survey.

1. Conclusion: the study found a low level of effective postnatal newborn care coverage, with a pro-rich distribution. Focus should be given on increasing institutional deliveries, expanding education, promoting ANC follow-up, improving wealth status, and newborns from vaginal deliveries. Furthermore, authorities and stakeholders should work on expanding health services in rural and affected regions to increase the population's access to newborn health services. (42-46)

The conclusion accurately identifies key determinants (e.g., wealth status, rural access) but remains vague on the 'how.' To strengthen the impact and utility of the findings for policymakers and stakeholders, the authors should provide more specific, actionable recommendations.

2. The postnatal newborn care includes: thermal protection (e.g., promoting skin-to-skin contact between mother and infant); hygienic umbilical cord and skin care; early and exclusive breastfeeding; screening for signs of serious health problems or need for additional care (e.g., low birth weight, sick, or Human immunodeficiency virus infected mother); and preventive treatment (e.g., Bacillus Calmette-Guérin vaccine (BCG) and Hepatitis B immunizations, vitamin K, and ocular prophylaxis). (93-98)

The summary of postnatal care practices is accurate; however, it currently lacks citations. To strengthen the manuscript and allow readers to verify the guidelines, please add references from established clinical guidelines (e.g., WHO, UNICEF, or relevant national bodies) for each of the points mentioned.

3. Most studies use crude coverage indicators, which are frequently used in maternal and child health studies, representing the tip of the iceberg because they only consider contact between women or children and health care providers. They are unable to show the level of adherence to care standards, the manner in which services are delivered, or the quality of care offered. (117-120)

The authors correctly identify the limitation of 'crude coverage' indicators. However, the manuscript must explicitly state how their own study's methodology addresses this gap. Please specify: What specific components of your 'effective coverage' metric directly measure quality of care, adherence to guidelines, or the process of service delivery, thereby moving beyond simple contact with a provider?

4. The introduction does not clearly define a concrete research gap. Please explicitly state the specific knowledge void that your study aims to fill.

5. This study included 4335 weighted sample women of reproductive age (15–49) who had given birth in the two years preceding the survey.

Please clarify the sampling procedure. How were the 4,335 participants selected from the larger survey? What were the specific eligibility criteria (inclusion/exclusion) for this particular analysis?

6. The authors state that the Erreygers normalized concentration index was used, and while the concentration curve graph is helpful for visualization, the precise numerical values are missing. For the sake of transparency, reproducibility, and to allow readers to ascertain the exact magnitude of inequality, please provide a table of the calculated Erreygers index values, either in the main manuscript or as a supplementary appendix.

7. All figures in the manuscript are currently missing a caption. This is a critical omission that must be corrected.

8. Authors have mentioned “Possible explanations for this significant variation could be the later study used a video-recorded observation, while this study used respondents’ responses only.” Please elaborate on this statement. How is this statement providing any justification?

9. The limitations section should acknowledge that the use of cross-sectional data prevents the establishment of causal relationships between the identified factors (e.g., wealth, education) and the outcome of effective postnatal care. Please add this standard but critical limitation.

10. The manuscript lacks a dedicated section on policy recommendations. Based on your key findings, specifically the pro-rich inequality and the determinants of effective care, please add clear, actionable recommendations for policymakers and stakeholders.

Reviewer #2: Firstly, I would like to commend the authors on their well-structured and scientifically rigorous study. I have identified a few areas where the manuscript could be improved to enhance its clarity and impact. Please find my specific comments below:

1) The introduction lacks a clear structure and logical flow. The research gaps and research questions are unclear. I suggest the authors to set a context by explaining why studying wealth-based inequality in effective coverage of postnatal newborn care is important in Tanzania.

2) It is better to rewrite based a) what the issue is, b) why it is important, c) what the literature says, d) what the research gap (Theoretical knowledge, empirical, evidence, population, methodological gaps, etc.) and, e) what the question in this study are, f) objectives of the study or how this study could contribute to the literature.

3) Yet, to be of interest for readers outside Tanzania, too, the paper should also refer to some studies in the international literature, to highlight the gaps and unanswered questions, the methodologies used, etc. This could also help with the interpretation of the results, as it could allow international comparisons.

4) The authors have tried to explained Wealth-related inequality in effective coverage of postnatal newborn care. Here household’s wealth is the exposure variables. The authors are trying to estimate factors contributing to the existing inequality across wealth class. How wealth categories can be taken as a factor in this decomposition model (Check table 3 and 4)?

5) In some part, the authors repeated the results. It would be more beneficial if the discussion primarily emphasizes the key findings, their interpretations, and the implications they carry for public health interventions and potential policy adjustments. For improved coherence and academic rigor, the discussion section may be organized into the following three sub-sections: Key Findings, Policy Recommendations, Strengths and Limitations. These additions would greatly enhance the overall quality and policy relevance of the manuscript.

6) In this case, one paragraph should encompass discussion on one key finding of the study. (The study may have several key findings to fulfil the research questions and objectives. Therefore, each paragraph will be dedicated for discussing one finding). In this paragraph the authors must justify this finding based on the previous study findings, including similarity and dissimilarity of this finding to other study findings. Need to justify why, how, you have got this finding.

7) Authors can also compare the results of different methods and assess which was more fruitful and why. Discuss the implications of this research and compare it to the results of other studies. Authors can cite related studies to show how your study compares (in addition to the introduction).

8) One paragraph on the policy recommendation. In this paragraph, the authors must highlight, how the findings of this study help the policy makers to develop new policies to improve the current situation and to solve this problem based on this current study findings, or how the study findings will help the policymakers to modify existing policies in this field.

**********

what does this mean?). If published, this will include your full peer review and any attached files.

If you choose “no”, your identity will remain anonymous but your review may still be made public.

**Do you want your identity to be public for this peer review?**  For information about this choice, including consent withdrawal, please see our Privacy Policy

Reviewer #1: No

Reviewer #2: No

**********

[NOTE: If reviewer comments were submitted as an attachment file, they will be attached to this email and accessible via the submission site. Please log into your account, locate the manuscript record, and check for the action link "View Attachments". If this link does not appear, there are no attachment files.]

To ensure your figures meet our technical requirements, please review our figure guidelines: https://journals.plos.org/plosone/s/figures

You may also use PLOS’s free figure tool, NAAS, to help you prepare publication quality figures: https://journals.plos.org/plosone/s/figures#loc-tools-for-figure-preparation.

NAAS will assess whether your figures meet our technical requirements by comparing each figure against our figure specifications.

Attachment Submitted filename: PLOS one.docx

# Author comment: Author response to Decision Letter 1

**DOI:** 10.1371/journal.pone.0358220.r002

29 Jan 2026

Responses to the reviewers' comments are provided in the 'Response to reviewers' document. Please refer to that document. Thank you.

Attachment Submitted filename: Response to reviewers.docx

# Peer review: Decision Letter 1

**DOI:** 10.1371/journal.pone.0358220.r003

**Authors:** Pritam Ghosh

22 Apr 2026

Dear Dr. Gebrekidan,

Thank you for submitting your manuscript to PLOS ONE. After careful consideration, we feel that it has merit but does not fully meet PLOS ONE’s publication criteria as it currently stands. Therefore, we invite you to submit a revised version of the manuscript that addresses the points raised during the review process.

Please submit your revised manuscript by Jun 06 2026 11:59PM. If you will need more time than this to complete your revisions, please reply to this message or contact the journal office at plosone@plos.org. When you're ready to submit your revision, log on to https://www.editorialmanager.com/pone/ and select the 'Submissions Needing Revision' folder to locate your manuscript file.

- A letter that responds to each point raised by the academic editor and reviewer(s). You should upload this letter as a separate file labeled 'Response to Reviewers'.
- A marked-up copy of your manuscript that highlights changes made to the original version. You should upload this as a separate file labeled 'Revised Manuscript with Track Changes'.
- An unmarked version of your revised paper without tracked changes. You should upload this as a separate file labeled 'Manuscript'.

If you would like to make changes to your financial disclosure, please include your updated statement in your cover letter. Guidelines for resubmitting your figure files are available below the reviewer comments at the end of this letter.

If applicable, we recommend that you deposit your laboratory protocols in protocols.io to enhance the reproducibility of your results. Protocols.io assigns your protocol its own identifier (DOI) so that it can be cited independently in the future. For instructions see: https://journals.plos.org/plosone/s/submission-guidelines#loc-laboratory-protocols. Additionally, PLOS ONE offers an option for publishing peer-reviewed Lab Protocol articles, which describe protocols hosted on protocols.io. Read more information on sharing protocols at https://plos.org/protocols?utm_medium=editorial-email&utm_source=authorletters&utm_campaign=protocols.

As the corresponding author, your ORCID iD is verified in the submission system and will appear in the published article. PLOS supports the use of ORCID, and we encourage all coauthors to register for an ORCID iD and use it as well. Please encourage your coauthors to verify their ORCID iD within the submission system before final acceptance, as unverified ORCID iDs will not appear in the published article. *Only*  the individual author can complete the verification step; PLOS staff *cannot*  verify ORCID iDs on behalf of authors.

We look forward to receiving your revised manuscript.

Kind regards,

Pritam Ghosh, Ph. D.

Academic Editor

PLOS One

**Journal Requirements:**

If the reviewer comments include a recommendation to cite specific previously published works, please review and evaluate these publications to determine whether they are relevant and should be cited. There is no requirement to cite these works unless the editor has indicated otherwise.

**Additional Editor Comments:**

The authors have made commendable efforts to upgrade the manuscript. I have identified a few areas where the manuscript could be improved to enhance its clarity and impact. Please find my specific comments below:

1. The research gaps and research questions are unclear. The Introduction section should be reorganised to follow a more standard academic structure:

(a) a clear presentation of the context and background of the study;

(b) a focused literature review that synthesises existing evidence, identifies research gaps (Theoretical, Knowledge, empirical, evidence, population, methodological gap etc.), and culminates in clearly articulated research questions; and

(c) a concluding paragraph that explicitly outlines the research objectives.

2. Discussion section must be improved dramatically. In some part the authors repeated the results. It would be more beneficial if the discussion primarily emphasized the key findings, their interpretations, and the implications they carry for public health interventions and potential policy adjustments. For improved coherence and academic rigor, the discussion section may be organized into the following three sub-sections: Key Findings, Policy Recommendations, Limitations and Strengths. These additions would greatly enhance the overall quality and policy relevance of the manuscript.

3. Each major finding should be discussed in a separate paragraph. Since the study likely addresses multiple research questions and objectives, devote one paragraph to each key finding. In these paragraphs, the authors should justify the results with reference to previous studies— (a) highlighting both similarities and differences. (b) Additionally, explain why and how the observed findings emerged in this study.

Reviewer 3

Title: Wealth-related inequality in effective coverage of postnatal newborn care in Tanzania; A decomposition analysis of the 2022 Tanzania Demographic and Health Survey.

Thank you for entrusting me with the opportunity to review the manuscript for your esteemed journal. After conducting a thorough and critical review, I would like to highlight several findings that require attention. I recommend accepting this article once the necessary corrections have been implemented.

Minor comments:

1. Revised the title as “Wealth-related inequality in effective coverage of postnatal newborn care in Tanzania: A decomposition analysis of the Tanzania Demographic and Health Survey”

2. Methods section of the abstract need to be revised as structural issues exists.

3. Line 318 “Obstetrics realted characteristics of study participants” revised with “Obstetrics related characteristics of study participants” and title of the Table 2.

4. Include ECI value in the table 4 or in separate table, with p-vale, unexplained/error.

5. It would be good if a inequality curve present based on wealth-based ECI value to reflects the inequality inequalities in effective newborn care coverage (revise).

6. Discussion needs to be revised which should not repeated the results only rather it should be mentioned the possible reasons and the factors of the outcomes. Citing only the research article also not be appropriate always, it should be discussed the results with the previous literatures.

7. Conclusion is too generic, and overstated; conclusion need to be condensed.

[Note: HTML markup is below. Please do not edit.]

Reviewers' comments:

Reviewer's Responses to Questions

**Comments to the Author**

Reviewer #1: All comments have been addressed

Reviewer #3: All comments have been addressed

**********

2. Is the manuscript technically sound, and do the data support the conclusions?

Reviewer #1: No

Reviewer #3: Yes

**********

3. Has the statistical analysis been performed appropriately and rigorously?

Reviewer #1: Yes

Reviewer #3: Yes

**********

4. Have the authors made all data underlying the findings in their manuscript fully available?

The PLOS Data policy

Reviewer #1: Yes

Reviewer #3: Yes

**********

5. Is the manuscript presented in an intelligible fashion and written in standard English?

Reviewer #1: Yes

Reviewer #3: Yes

**********

Reviewer #1: Wealth-related inequality in effective coverage of postnatal newborn care in Tanzania: A decomposition analysis of the 2022 Tanzania Demographic and Health Survey is a well-written manuscript. All comments have been addressed. The paper can be accepted for publication.

Reviewer #3: Thank you for entrusting me with the opportunity to review the manuscript for your esteemed journal. After conducting a thorough and critical review, I would like to highlight several findings that require attention. I recommend accepting this article once the necessary corrections have been implemented.

Minor comments:

1. Revised the title as “Wealth-related inequality in effective coverage of postnatal newborn care in Tanzania: A decomposition analysis of the Tanzania Demographic and Health Survey”

2. Methods section of the abstract need to be revised as structural issues exists.

3. Line 318 “Obstetrics realted characteristics of study participants” revised with “Obstetrics related characteristics of study participants” and title of the Table 2.

4. Include ECI value in the table 4 or in separate table, with p-vale, unexplained/error.

5. It would be good if a inequality curve present based on wealth-based ECI value to reflects the inequality inequalities in effective newborn care coverage (revise).

6. Discussion needs to be revised which should not repeated the results only rather it should be mentioned the possible reasons and the factors of the outcomes. Citing only the research article also not be appropriate always, it should be discussed the results with the previous literatures.

7. Conclusion is too generic, and overstated; conclusion need to be condensed.

**********

what does this mean?). If published, this will include your full peer review and any attached files.

If you choose “no”, your identity will remain anonymous but your review may still be made public.

**Do you want your identity to be public for this peer review?**  For information about this choice, including consent withdrawal, please see our Privacy Policy

Reviewer #1: No

Reviewer #3: **Yes:** Mriganka Dolui

**********

[NOTE: If reviewer comments were submitted as an attachment file, they will be attached to this email and accessible via the submission site. Please log into your account, locate the manuscript record, and check for the action link "View Attachments". If this link does not appear, there are no attachment files.]

To ensure your figures meet our technical requirements, please review our figure guidelines: https://journals.plos.org/plosone/s/figures

You may also use PLOS’s free figure tool, NAAS, to help you prepare publication quality figures: https://journals.plos.org/plosone/s/figures#loc-tools-for-figure-preparation.

NAAS will assess whether your figures meet our technical requirements by comparing each figure against our figure specifications.

Attachment Submitted filename: Comments.docx

# Author comment: Author response to Decision Letter 2

**DOI:** 10.1371/journal.pone.0358220.r004

2 Jun 2026

Response letter

Journal: PLOS One

Title: Wealth-related inequality in effective coverage of postnatal newborn care in Tanzania; A decomposition analysis of the 2022 Tanzania Demographic and Health Survey.

Submission ID: PONE-D-25-19119R1

Corresponding Author: Amanuel Yosef

Dear Pritam Ghosh, Ph. D. (Editor) and Reviewers,

We would like to forward our deepest gratitude for the constructive, helpful and insightful comments given to this manuscript by the editor and reviewers which are vital in improving the substance and contents of our manuscript. We considered each comment and clarify questions and comments raised by the reviewers on the manuscript thoroughly. Our point by-point responses for each comment and questions are described in detailed on the attached response letter. Further, the details of changes were shown by track changes in the supplementary document attached. Thank you. N.B. The page numbers and line numbers mentioned are for the revised manuscript with tracked changes.

Sincerely

Amanuel Yosef (corresponding author)

Attachment Submitted filename: Response_to_reviewers_auresp_2.docx

# Editor report: Decision Letter 2

**DOI:** 10.1371/journal.pone.0358220.r005

**Authors:** Pritam Ghosh

9 Jun 2026

Dear Dr. Amanuel Yosef Gebrekidan,

Thank you for submitting your manuscript to PLOS ONE. After careful consideration, we feel that it has merit but does not fully meet PLOS ONE’s publication criteria as it currently stands. Therefore, we invite you to submit a revised version of the manuscript that addresses the points raised during the review process.

Please submit your revised manuscript by Jul 24 2026 11:59PM. If you will need more time than this to complete your revisions, please reply to this message or contact the journal office at plosone@plos.org. When you're ready to submit your revision, log on to https://www.editorialmanager.com/pone/ and select the 'Submissions Needing Revision' folder to locate your manuscript file.

- A letter that responds to each point raised by the academic editor and reviewer(s). You should upload this letter as a separate file labeled 'Response to Reviewers'.
- A marked-up copy of your manuscript that highlights changes made to the original version. You should upload this as a separate file labeled 'Revised Manuscript with Track Changes'.
- An unmarked version of your revised paper without tracked changes. You should upload this as a separate file labeled 'Manuscript'.

If you would like to make changes to your financial disclosure, please include your updated statement in your cover letter. Guidelines for resubmitting your figure files are available below the reviewer comments at the end of this letter.

If applicable, we recommend that you deposit your laboratory protocols in protocols.io to enhance the reproducibility of your results. Protocols.io assigns your protocol its own identifier (DOI) so that it can be cited independently in the future. For instructions see: https://journals.plos.org/plosone/s/submission-guidelines#loc-laboratory-protocols. Additionally, PLOS ONE offers an option for publishing peer-reviewed Lab Protocol articles, which describe protocols hosted on protocols.io. Read more information on sharing protocols at https://plos.org/protocols?utm_medium=editorial-email&utm_source=authorletters&utm_campaign=protocols.

As the corresponding author, your ORCID iD is verified in the submission system and will appear in the published article. PLOS supports the use of ORCID, and we encourage all coauthors to register for an ORCID iD and use it as well. Please encourage your coauthors to verify their ORCID iD within the submission system before final acceptance, as unverified ORCID iDs will not appear in the published article. *Only*  the individual author can complete the verification step; PLOS staff *cannot*  verify ORCID iDs on behalf of authors.

We look forward to receiving your revised manuscript.

Kind regards,

Pritam Ghosh, Ph. D.

Academic Editor

PLOS One

Journal Requirements:

If the reviewer comments include a recommendation to cite specific previously published works, please review and evaluate these publications to determine whether they are relevant and should be cited. There is no requirement to cite these works unless the editor has indicated otherwise.

Please review your reference list to ensure that it is complete and correct. If you have cited papers that have been retracted, please include the rationale for doing so in the manuscript text, or remove these references and replace them with relevant current references. Any changes to the reference list should be mentioned in the rebuttal letter that accompanies your revised manuscript. If you need to cite a retracted article, indicate the article’s retracted status in the References list and also include a citation and full reference for the retraction notice.

Additional Editor Comments (if provided):

One minor correction is required. You have to chnage the title slightly.

Insearfd of following title:

"Wealth-related inequality in effective coverage of postnatal newborn care in Tanzania; A decomposition analysis of the Tanzania Demographic and Health Survey."

The authors need to add the year of the Tanzania Demographic and Health Survey.

I think it will be like following:

"Wealth-related inequality in effective coverage of postnatal newborn care in Tanzania; A decomposition analysis of the Tanzania Demographic and Health Survey, 2022"

[Note: HTML markup is below. Please do not edit.]

[NOTE: If reviewer comments were submitted as an attachment file, they will be attached to this email and accessible via the submission site. Please log into your account, locate the manuscript record, and check for the action link "View Attachments". If this link does not appear, there are no attachment files.]

To ensure your figures meet our technical requirements, please review our figure guidelines: https://journals.plos.org/plosone/s/figures

You may also use PLOS’s free figure tool, NAAS, to help you prepare publication quality figures: https://journals.plos.org/plosone/s/figures#loc-tools-for-figure-preparation.

NAAS will assess whether your figures meet our technical requirements by comparing each figure against our figure specifications.

# Author comment: Author response to Decision Letter 3

**DOI:** 10.1371/journal.pone.0358220.r006

16 Jul 2026

Dear Editor, we appreciate the time and effort given to reviewing our paper. Based on your suggestion, we have amended the title. Please refer to the title of the manuscript. Thank you.

Attachment Submitted filename: Response_to_reviewers_auresp_3.docx

# Editor report: Decision Letter 3

**DOI:** 10.1371/journal.pone.0358220.r007

**Authors:** Pritam Ghosh

30 Aug 2026

Wealth-related inequality in effective coverage of postnatal newborn care in Tanzania; A decomposition analysis of the Tanzania Demographic and Health Survey, 2022.

PONE-D-25-19119R3

Dear Dr. Gebrekidan,

We’re pleased to inform you that your manuscript has been judged scientifically suitable for publication and will be formally accepted for publication once it meets all outstanding technical requirements.

Within one week, you’ll receive an e-mail detailing the required amendments. When these have been addressed, you’ll receive a formal acceptance letter and your manuscript will be scheduled for publication.

An invoice will be generated when your article is formally accepted. Please note, if your institution has a publishing partnership with PLOS and your article meets the relevant criteria, all or part of your publication costs will be covered. Please make sure your user information is up-to-date by logging into Editorial Manager at Editorial Manager® and clicking the ‘Update My Information' link at the top of the page. For questions related to billing, please contact billing support.

If your institution or institutions have a press office, please notify them about your upcoming paper to help maximize its impact. If they’ll be preparing press materials, please inform our press team as soon as possible -- no later than 48 hours after receiving the formal acceptance. Your manuscript will remain under strict press embargo until 2 pm Eastern Time on the date of publication. For more information, please contact onepress@plos.org.

Kind regards,

Pritam Ghosh, Ph. D.

Academic Editor

PLOS One

# Editor report: Acceptance letter

**DOI:** 10.1371/journal.pone.0358220.r008

**Authors:** Pritam Ghosh

PONE-D-25-19119R3

PLOS One

Dear Dr. Gebrekidan,

I'm pleased to inform you that your manuscript has been deemed suitable for publication in PLOS One. Congratulations! Your manuscript is now being handed over to our production team.

At this stage, our production department will prepare your paper for publication. This includes ensuring the following:

* All references, tables, and figures are properly cited

* All relevant supporting information is included in the manuscript submission,

* There are no issues that prevent the paper from being properly typeset

You will receive further instructions from the production team, including instructions on how to review your proof when it is ready. Please keep in mind that we are working through a large volume of accepted articles, so please give us a few days to review your paper and let you know the next and final steps.

Lastly, if your institution or institutions have a press office, please let them know about your upcoming paper now to help maximize its impact. If they'll be preparing press materials, please inform our press team within the next 48 hours. Your manuscript will remain under strict press embargo until 2 pm Eastern Time on the date of publication. For more information, please contact onepress@plos.org.

You will receive an invoice from PLOS for your publication fee after your manuscript has reached the completed accept phase. If you receive an email requesting payment before acceptance or for any other service, this may be a phishing scheme. Learn how to identify phishing emails and protect your accounts at https://explore.plos.org/phishing.

If we can help with anything else, please email us at customercare@plos.org.

Thank you for submitting your work to PLOS One and supporting open access.

Kind regards,

PLOS One Editorial Office Staff

on behalf of

Dr. Pritam Ghosh

Academic Editor

PLOS One
